# Comparing image quality of synchrotron and laboratory nano-CT scans: a round robin study

**DOI:** 10.1107/S160057752600086X

**Published:** 2026-02-20

**Authors:** Simon Wittl, Simon Zabler, Jonas Fell, Julie Villanova, Pierre Lhuissier, Stefanie Hildebrandt, Hans-Georg Herrmann

**Affiliations:** a Technology Campus Plattling Deggendorf Institute of Technology Bavaria Germany; b Application Center for CT in Metrology Fraunhofer IIS Bavaria Germany; c Core Facility for Correlative Microscopy and Tomography CoMiTo Saarland University Saarbruecken Saarland Germany; d Lightweight Systems Saarland University Saarbruecken Saarland Germany; e ID16B European Synchrotron Radiation Facility Grenoble France; f SIMaP Universite Grenoble Alpes Grenoble France; g Materials Synthesis and Development Fraunhofer Institute for Ceramic Technologies and Systems IKTS Dresden Saxony Germany; h Fraunhofer Institute for Nondestructive Testing IZFP Saarbruecken Saarland Germany; Paul Scherrer Institut, Switzerland

**Keywords:** nano-CT, synchrotron radiation, modulation transfer function

## Abstract

This study presents a comprehensive comparison of image quality between synchrotron-based and laboratory-based nano-computed tomography (nano-CT) systems, using a standardized 3D test phantom composed of monodisperse silica spheres. A round-robin approach was employed across multiple international facilities to benchmark performance across various imaging modalities and setups, including absorption and phase contrast techniques. Image quality was quantitatively assessed using three-dimensional signal-to-noise ratio (SNR_3D_), detection effectiveness (DE_3D_) and modulation transfer function (MTF_3D_) metrics. The results reveal that, while synchrotron-based nano-CT consistently delivers superior spatial resolution and shorter scan times, laboratory-based systems demonstrate competitive image quality at the cost of extended acquisition durations. Beyond the experimental comparison, the main contribution of this work is a standardized, open-source analysis framework that quantifies nano-CT image quality using SNR_3D_, DE_3D_ and MTF_3D_ of a specific phantom. This combination of metrics provides a reproducible basis for cross-platform benchmarking of synchrotron and laboratory nano-CT implementations and can be readily applied to future instruments.

## Abstract 2

A multi-facility round-robin comparison of synchrotron- and laboratory-based nano-computed tomography using a standardized 3D phantom reveals superior resolution and acquisition speed at synchrotrons, while advanced laboratory systems achieve competitive image quality with extended scan times; phase contrast modalities further enhance structural visibility.

## 1. Introduction

By X-ray nano-computed tomography (nano-CT) we designate experiments or devices which allow for mapping an object’s X-ray optical density to three-dimensional coordinates with a precision of 400 nm or better (Zabler *et al.*, 2021 ▸). This choice appears arbitrary at first, particularly because early generations of X-ray microscopes did not fulfill this criterion (Lai *et al.*, 1992 ▸). Nevertheless, recent investigations of sub-micrometre CT scanners reveal that the resolving power of this instrument class reaches down to 0.42 µm as verified by SNR^2^ (where SNR is the signal-to-noise ratio) and the modulation transfer function (MTF) of various CT scanners (Zabler *et al.*, 2020 ▸). Performing 3D CT scans with even higher precision introduces a new class of CT instruments, necessitating far better mechanical stability, in some cases optical devices and very long exposure times, if the instrument is not situated at synchrotron facilities.

Nano-CT has evolved from X-ray microscopy (XRM) which was pioneered at synchrotron beamlines in the late 1980s (Kirz & Rarback, 1985 ▸; Rarback *et al.*, 1988 ▸). Today, XRM exists in many variants: zone-plate microscopes, Kirkpatrick–Baez microscopes, full field- or scanning microscopes, or lens-less microscopy.

Zone-plate microscopes combine high-aspect-ratio Fresnel zone-plates (FZPs) with high-resolution X-ray cameras. The standard fabrication method of FZPs is electron beam lithography (EBL). The concept dates to Janos Kirz who proposed to use phase shifting FZPs instead of opaque zones (Kirz, 1974 ▸). The FZP’s diffraction efficiency η is a critical parameter when designing microscopes for a given wavelength (note, FZPs imply monochromatic imaging) (Jacobsen *et al.*, 1991 ▸),
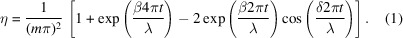
Here, *m* is the diffraction order, *n* = 1 − δ + *i*β the refractive index of the FZP material for a given wavelength λ, while *t* is the material thickness. Obviously, high-*Z* materials such as gold, tungsten or tantalum are preferred for their refractive power. Meanwhile, the resolving power of a first order FZP (which implies aperture matching condenser optics) is primarily limited by numerical aperture NA according to
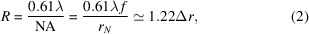
with *r*_*N*_ the outermost zone radius, *f* the focal length and Δ*r* the outermost zone width of the FZP. Equation (2) states that, in order to achieve a 50 nm focus, one needs at least 40 nm outermost zone width. Yet, even for gold to achieve the maximum possible diffraction efficiency at 8.6 keV energy according to equation (1), a thickness of 1.66 µm is required, hence an aspect ratio of 41.5:1 which is beyond the limits of EBL (Lai *et al.*, 1992 ▸).

While ideally the FZP material is shifting the X-rays by π or attenuates them completely, the efficiency of high resolution FZP for hard X-rays is significantly lower due to manufacturing limitations. By using an FZP of 0.9 µm gold and 100 nm outermost zone width, Yun *et al.* reported a focal spot of 150 nm full width at half-maximum (FWHM) at 8 keV in first order and 90 nm in third order (Yun *et al.*, 1999 ▸). Using a similar design but an outermost zone width of 50 nm in a FZP made of 0.5 µm thick tantalum, Suzuki *et al.* reported a 58 nm FWHM in first and 30 nm in third order diffraction spots at 8 keV (Suzuki *et al.*, 2005 ▸). Scanning a knife edge coated with an X-ray fluorescent metal across the focal plane became a common routine for validating these benchmarks. Smaller focal spots came at the cost of the FZP’s diffraction efficiency which was down to 5% at that time.

Higher aspect ratio FZPs could be produced by consecutive sputtering transparent and opaque materials onto a cylindrical core and cutting thick slices of the resulting layered structure. Awaji *et al.* used a sputtered-sliced SS-FZP with 25 keV photon energy on the 47XU-beamline at SPring-8 (Awaji *et al.*, 2002 ▸). Despite achieving only 500 nm spatial resolution, their study demonstrated the advantage of Zernike-type phase contrast over attenuation-based XRM. Feng *et al.* attempted to overcome the aspect ratio limit to increase diffraction efficiency by stacking identical zone lenses on top of one another (Feng *et al.*, 2007 ▸). David *et al.* came up with a similar idea, using a two-sided FZP to double the aspect ratio (Jefimovs *et al.*, 2007 ▸). Another approach for producing higher aspect ratios lies in fabricating one-dimensional zones by consecutive layer deposition, so-called multilayer Laue-lenses (MLLs). MLLs were first proposed by Joerg Maser in 2004 (Maser *et al.*, 2004 ▸). The idea was later adopted by Salditt *et al.* who achieved a sub-5 nm focus at 7.9 keV on PETRAIII’s P10 beamline (Döring *et al.*, 2013 ▸).

In 2005, Hignette *et al.* reported on XRM with a pair of bent graded multilayer X-ray mirrors [called Kirkpatrick–Baez (KB) optics], which he used, instead of an FZP, to focus 20.5 keV synchrotron X-rays into a 86 nm × 83 nm spot (Hignette *et al.*, 2005 ▸). Soon Mokso *et al.* turned this device into a nano-CT experiment, making use of spatial coherence (*i.e.* the very low angular divergence of synchrotron beams) to allow optical propagation across extended object–detector distances (Mokso *et al.*, 2007 ▸). Propagation-based phase contrast (PBPC) typically requires four CT scans from the same view but at slightly different geometric magnification, hence propagation length, for numerical phase retrieval prior to volume image reconstruction. This holotomography scheme as well as the phase retrieval algorithm stem from well established synchrotron sub-micrometre tomography pioneered at the European synchrotron (Cloetens *et al.*, 1999*b* ▸). Likewise, Zernike-type phase contrast requires numerical phase retrieval prior to volume image reconstruction.

With nanotomography, spatial resolution could be assessed from volume images directly (*e.g.* line profile across some material interface). Unlike knife-edge scans of the focal spot, nano-CT images displayed 290 nm image blur which equated to 3.2 pixels in the object domain (Mokso *et al.*, 2007 ▸). On the same device, Requena *et al.* reported a smallest visible detail of 180 nm width (Requena *et al.*, 2009 ▸). In 2018, Larsson *et al.* designed a phantom of nanoporous gold for estimating the resolving power of nano-CT scans, specifically by computing the Fourier-shell correlation (FSC) from nano-CT scans (Larsson *et al.*, 2019 ▸). Scanning this phantom with an FZP-microscope on P05 beamline at PETRA III led to an estimate of 62 nm (∼3.1 pixel with 20 nm pixel^−1^ sampling, 80 nm outermost zone width and 14 keV photons). Flenner *et al.* used a similar FZP (*r* = 300 µm, Δ*r* = 50 nm, *f* = 113.1 mm at 11 keV) to transform P05’s quasi-parallel synchrotron beam into a cone emerging from a virtual focal spot of 95 nm size. Placing the object 168 mm downstream of their order sorting aperture and the detector at 21 m results in strongly magnified (125×) and partially coherent projections. After phase retrieval and tomographic back-projection, the authors reported 176 nm 3D resolution at 52 nm object sampling (Flenner *et al.*, 2020 ▸).

Since 2008, nano-CT experiments have increasingly used lens-less imaging techniques, with ptychography being the most prominent (Dierolf *et al.*, 2008 ▸). Like Fourier transform holography, ptychography consists of scanning the object with a small pinhole which creates fully coherent illumination while the detector is recording the far-field holographic intensities (Eisebitt *et al.*, 2004 ▸). Holler reported 16 nm volumetric resolution with ptychography of porous silica at 6.2 keV at the Swiss Light Source (6.9 nm pixel^−1^ sampling) (Holler *et al.*, 2014 ▸).

Using incoherent laboratory X-ray sources, at least two nano-CT experiments have emerged from the previously described development which took place at synchrotron light sources. The X-ray shadow microscope (the commercial name is XRM-NanoCT by the company Procon X-ray) stems from the original design by Manfred von Ardenne who proposed to convert electron microscopes into X-ray microscopes by focusing the electron beam onto metal targets (von Ardenne, 1939 ▸). In 2003, Mayo *et al.* built such a device which puts a copper anode inside a scanning electron microscope (SEM) thus converting the latter into a sub-micrometre CT scanner, reporting 0.56 µm resolution (Mayo *et al.*, 2003 ▸; Mayo *et al.*, 2007 ▸). In 2021, Lutter *et al.* reported 150 nm spatial resolution for nano-CT scans from a similar device, using a nanometre-sharp etched tungsten tip instead of a copper anode, hence polychromatic X-rays (*U*_A_ = 30 kV) (Lutter *et al.*, 2021 ▸).

A transmission X-ray microscope (TXM) with FZP for focusing and an elliptical mono-capillary as condenser optic was introduced by Tkachuk *et al.* in 2006 who reported 50 nm resolution from nano-CT scans of integrated circuits (Tkachuk *et al.*, 2006 ▸). Like synchrotron TXM, their images were monochromatic, hence these devices use either copper *K*α (8.05 keV) or chromium *K*α (5.4 keV) high power X-ray anodes. Since 2013, nano-CT scanners have been built by X-radia Inc. but commercialized by Carl Zeiss AG. In 2012, Epting *et al.* reported 50 nm spatial resolution on scans from fuel cell electrodes using copper *K*α radiation (Epting *et al.*, 2012 ▸).

While nano-CT experiments are highly diverse with regard to their instrumentation and even data processes, the resulting scans display a high homogeneity in terms of image sampling, material contrast and data size. From a data scientist point of view it therefore makes sense to compare different experiments based on a highly homogeneous dataset using one structured material and universal image quality metrics.

Nano-CT scans are volumetric images showing the object’s X-ray optical density (attenuation and/or refraction). While fine spatial sampling (assuming isotropic, hence cubic voxels) is a requirement for observing structures of a certain size, spatial resolution generally falls short of voxel sampling and needs to be verified for a certain object detail/material interface.

Generally, in CT imaging, it is noise (in addition to blur) which is obscuring structural detail. Consequently, assessing spatial resolution in terms of modulation transfer (MTF or FSC) needs to be complemented by measurements of signal-to-noise (SNR) or contrast-to-noise (CNR) ratio. When the former is measured as a radial power spectrum, the nominator of SNR^2^(*u*) = S^2^(*u*)/N^2^(*u*) comprises material contrast, MTF and object shape, while the denominator scales with noise power.

Phase contrast arising from propagation (*e.g.* in absorption-mode TXM under slight FZP defocus) produces Fresnel fringes that cause the bright rims around the spheres and the comparatively higher intensity of the smaller spheres. Note that Zernike phase-contrast puts a phase-shifting mask behind the FZP for generating Zernike-type phase contrast. This phase ring does not yield fringes similar to propagation based phase contrast, so *H*_phase_ ≃ 1.

With phase contrast invoked, image reconstruction must include a certain phase-retrieval process either by image filtering or by numerically solving analytical equations. Phase-retrieval is frequently, but not always, applied prior to tomographic back projection (BP). Unlike micro- and sub-micro-CT, nano-CT achieves BP generally by iterative reconstruction schemes, such as SIRT or SART (Sidky & Pan, 2008 ▸). The latter allow for including error-correcting steps, such as object self-alignment and de-noising, *e.g.* by minimizing total variation, alternating with BP of ray sums. Calculating the image residuals after each step requires forward projecting the volume image, sometimes including phase contrast and modeling the recording device. In summary, nano-CT volume image reconstruction is adapted to a single experimental setting. While having a critical influence on the resulting image quality, BP cannot be modeled by a linear filter. Consequently, comparing nano-CT image quality must regard image acquisition and BP as one process while keeping in mind that quality improvement may result equally from changing experimental conditions or image processes.

Note that this study investigates nano-CT experiments operating at 0.23 nm wavelength (chromium *K*α) and below. The authors intentionally excluded nano-CT experiments operating in the soft X-ray regime, *i.e.* up to 4.5 nm, or carbon *K*α, and even in EUV (up to 121 nm, or hydrogen *K*α). Such settings are frequently applied to scan biological structures and have the object in vacuum. Experimental conditions therefore resemble electron microscopy rather than hard X-ray imaging which takes place under ambient conditions. The exception is the XRM shadow microscope which has the object under vacuum, despite using energies from 5 keV to 30 keV.

## 2. Materials and method

### 2.1. Laboratory-based nano-CT

The instrument names, the institutes and facilities participating in this study are listed in Table 1 ▸. Additionally, the equipment of the systems along with the scan parameters are listed in Table 2 ▸. A detailed description of all the systems is provided in this section.

#### 2.1.1. Zeiss Xradia 810 Ultra

This FZP-based X-ray microscope is equipped with a 5.4 keV rotating anode Cr-source and a Zernike phase ring for positive phase contrast imaging allowing imaging of 16 µm × 16 µm large areas with object pixel sampling of 16 nm. The optical magnification by the FZP is ∼10, hence the images are recorded by a lens-coupled indirect detector (LCID) which is sampling a thin-film scintillator with ∼320 nm pixel^−1^. Phase retrieval and volume image reconstruction are not documented and therefore cannot be cited in this study.

The 810 Ultra microscope often uses gold marker spheres to facilitate the correction of geometric misalignments.

#### 2.1.2. ProCon XRM-II nanoCT

The shadow microscope XRM-II nanoCT (ProCon X-Ray, Sarstedt, Germany) is based on a Jeol JSM-7900F scanning electron microscope (Jeol, Tokyo, Japan) and operated by the core facility for Correlative Microscopy and Tomography (CoMiTo) at Saarland University.

Inside the vacuum chamber the SEM-based CT is equipped with a second sample stage (Nanolathe by Kleindiek Nanotechnik GmbH, Reutlingen, Germany) which facilitates rotation about an arbitrarily defined region of interest (eucentric rotation) with a concentricity of less than 500 nm. Meanwhile an additional manipulator carries the anode/X-ray target which can be an etched metal needle target (NT, typically tungsten with about 30 nm tip diameter), a foil or a bulk target (BT, *e.g.* platinum). The geometry and chemical composition of the target can be tailored to specifically influence the focal spot size, the emitted X-ray spectrum, and the overall X-ray intensity, allowing for controlled adjustment of image resolution and contrast. Target and object are set in very close proximity to yield geometric magnifications *M* > 1000. The X-ray detector, a photon counting CdTe sensor, model WidePIX-5x2 (ADVACAM, Praha, Czech Republic), is set at a fixed focus–detector distance (FDD ≃ 40 cm) flanged to the outside of a beryllium exit window (Fell *et al.*, 2023*b* ▸; Fell *et al.*, 2023*c* ▸).

Throughout the scanning process, the electron gun operates at an acceleration voltage of 30 keV, focusing the electrons onto the X-ray target, where their interaction induces the emission of photons utilized for X-ray imaging. The lower detector threshold was set to 5 keV to extinguish thermal noise. Volume image reconstruction uses SART with incremental geometry correction [self alignment, see Mayo *et al.* (2007 ▸)]. Paganin-type phase retrieval and Wiener deconvolution are applied in one step to the reconstructed volume image (Ullherr & Zabler, 2015 ▸). Further information about the method and the system can be found in Lutter *et al.* (2021 ▸) and Fell *et al.* (2023*a* ▸).

### 2.2. Synchrotron

#### 2.2.1. SPring-8

Two beamlines of Japan’s SPring-8 synchrotron contributed scans to this study: 47XU and 20XU. During multi-bunch mode, SPring-8 delivers a ring current of 100 mA from 8 GeV storage electrons. The full-field X-ray microscope at BL47XU is situated 45 m downstream of an in-vacuum undulator source whose first harmonic was set to 8 keV and monochromated with a silicon double crystal mirror for the present tests. In the case of BL47XU, the distance between object and detector is limited to <7 m, yielding optical magnifications by FZP *M* = 30–70 for X-ray energies up to 10 keV. TXM images can be recorded in attenuation or Zernike phase contrast (Awaji *et al.*, 2002 ▸).

The beamline BL20XU is located 248 m downstream of the undulator source. It is used for various experiments: microbeam, scanning microscopy, imaging microscopy, holography, phase-contrast micro-tomography, interferometry, and ultra-small angle scattering (Suzuki *et al.*, 2004 ▸). For the present scans the photon energy was set to 30 keV.

High-energy X-ray nano-CT at BL20XU uses two experimental hutches separated by 165 m, yielding optical magnifications of *M* = 150–300 at X-ray energies 20–37.7 keV (Takeuchi & Suzuki, 2020 ▸). The large area condenser zone plate (CZP) provides very high photon flux (*e.g.* for *in situ* nano-CT) while allowing for a field of view of 100 µm in diameter. 3D image resolution <100 nm have been reported for both beamlines.

#### 2.2.2. ESRF ID16B

The ID16B endstation of the European synchrotron is designed for nano-imaging as well as nano-scanning microscopy (Martínez-Criado *et al.*, 2016 ▸). Two elliptically bent multilayer-coated silicon mirrors are focusing X-rays of a tunable energy to a virtual monochromatic spot of 50 nm × 50 nm size. After the recent upgrade, a nano-CT scan merely takes 1 min time, making ID16B the instrument of choice for time resolved nanotomography (Villanova *et al.*, 2017 ▸).

While the working distance between the second mirror and the focal spot is only ∼30 mm, the focus–detector distance for the present tests was 705 mm thus allowing for strong geometric magnification and significant optical propagation. The detector is an LCID with a PCO edge-4.2 sCMOS camera photographing a 30 µm thin LSO scintillator crystal through a 10× magnifying tandem lens, hence with 0.65 µm pixel^−1^ screen sampling. Sampling the object with 25 nm pixel^−1^ in this configuration requires *M* = 26 geometric magnification, which yields *d*_eff_ = 26 mm propagation length.

The present scans were recorded at 29.6 keV photon energy. Note that, for comparison, the experiment was repeated at 17.5 keV. Object sampling was 50 nm, 25 nm and 15 nm. In each setting four full scans were acquired, each comprising 2301 views of 10 ms exposure, recorded at four slightly different propagation distances, hence different geometric magnifications with the target sampling corresponding to the highest magnification. Prior to tomographic back projection, iterative phase retrieval combines the four Fresnel-propagated radiographs to yield a single phase projection view of the object with the target pixel sampling (Yu *et al.*, 2018 ▸). Faint displacements of the field of view, caused for example by thermo-mechanical drift of the instrument, are also compensated for during the phase retrieval procedure. Such acquisitions are referred to as multi-distance holotomography scans (Zabler *et al.*, 2005 ▸). For the ESRF data, the performance of various phase-retrieval algorithms and the impact of the Paganin parameter *p* are evaluated in Appendix *A*[App appa].

#### 2.2.3. DESY PETRA III P05

P05 is an imaging beamline in the Max-von-Laue hall of the Hamburg 6 GeV storage ring PETRA III which began operations in 2013. In multi-bunch operation the ring carries a 100 mA electron current. The P05 beamline uses an undulator source and comprises two experimental hutches which are distanced by 20 m along the beam: EH1 being at 65–69 m, and EH2 81–89 m downstream of the undulator source. When operating FZP-based ‘nano-holotomography’ the setup uses both hutches yielding object–detector distances of 16–22 m (Flenner *et al.*, 2020 ▸). In this setup, a 300 µm wide FZP creates a virtual focal spot at 133 mm focal distance. An order sorting aperture takes out unfocused light thus creating an 11 keV quasi-monochromatic cone beam. The X-ray camera in EH2 is a Hamamatsu CC12849-101U with 6.5 µm pixels tapered to a 10 µm GOS scintillator. For the present tests, the object was sampled at a single source–object distance with 56.2 nm pixel^−1^. With the detector in EH2, virtual focus–object distances range between 138 mm and 190 mm, *i.e.* propagation distances *d* of similar magnitude. Vacuum pipes between object and camera reduce air scattering and absorption.

### 2.3. 3D SNR and detection effectiveness (DE) of volume images

The 3D power spectrum *D*_3D_(*u*) is defined as the radial average of the squared magnitude of the 3D Fourier transform of the reconstructed volume. The noise power spectrum *N*_3D_(*u*) is obtained by fitting a noise model to the high-frequency region of *D*_3D_(*u*), where the structural signal is negligible. The structural signal is then *S*_3D_(*u*) = *D*_3D_(*u*) − *N*_3D_(*u*). Hereby, *u* = 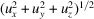 represents the radial spatial frequency assuming that the imaging properties of CT scanners are mostly isotropic. Thus, SNR_3D_ is obtained,
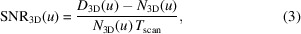
where *T*_scan_ is the total scan time. Modeling the signal power spectrum (PS) is achieved through computation of an ideal PS of the object structure *S*_object_(*u*). The latter is basically a noiseless version of the phantom whereby perfectly round and sharply contrasted spheres of the measured size are placed at the center coordinates of each detected sphere (volume processing explained below).

Normalizing SNR_3D_ with respect to *S*_object_(*u*) ideally cancels out the structural signal component of our sphere phantom. The resulting, more general, quantity is detection effectiveness,
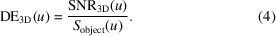
Multiplied with the structure of an arbitrary object (made of SiO_2_), DE yields a quantitative prediction of its signal-to-noise PS. To improve the smoothness of the DE_3D_(*u*) curves, the used SNR_3D_(*u*) can alternatively be computed directly from the modeled signal power spectrum *S*_3D_(*u*), rather than as the residual between the total power *D*_3D_(*u*) and the estimated noise *N*_3D_(*u*). Additionally, the DE was scaled using the number of detected spheres in the volume, compensating for fluctuations in sphere count across different datasets.

### 2.4. Modeling modulation transfer in CT images

**Figure 1:**
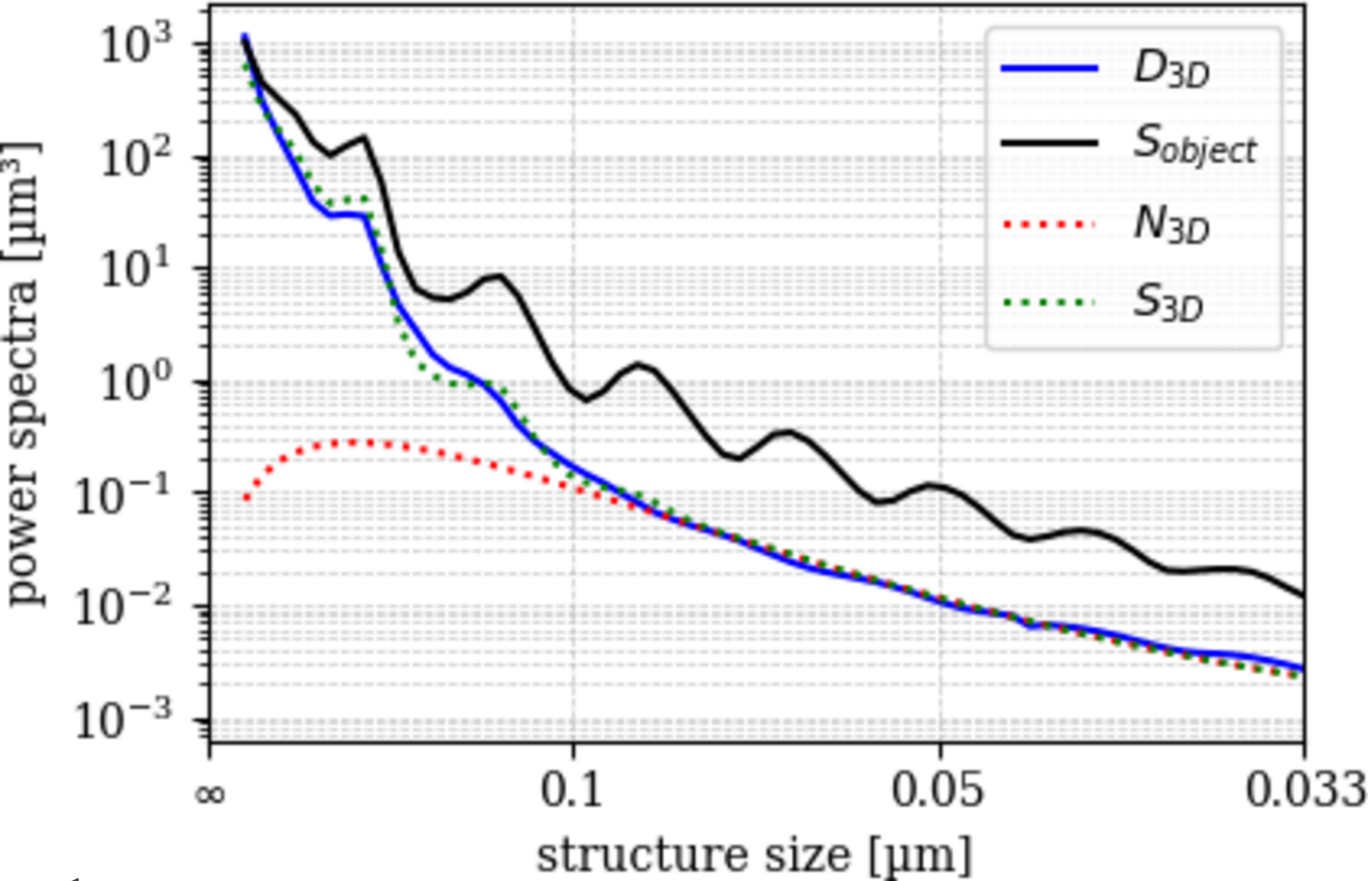
Radial power spectra of the simulated data (*S*_object_), scan data (*D*_3D_) and noise model (*N*_3D_). Peaks indicate periodicity of the signal. The shown data are from the ‘esrf: id16b - 29.5kev - holo - 25 nm’ scan.

**Figure 2:**
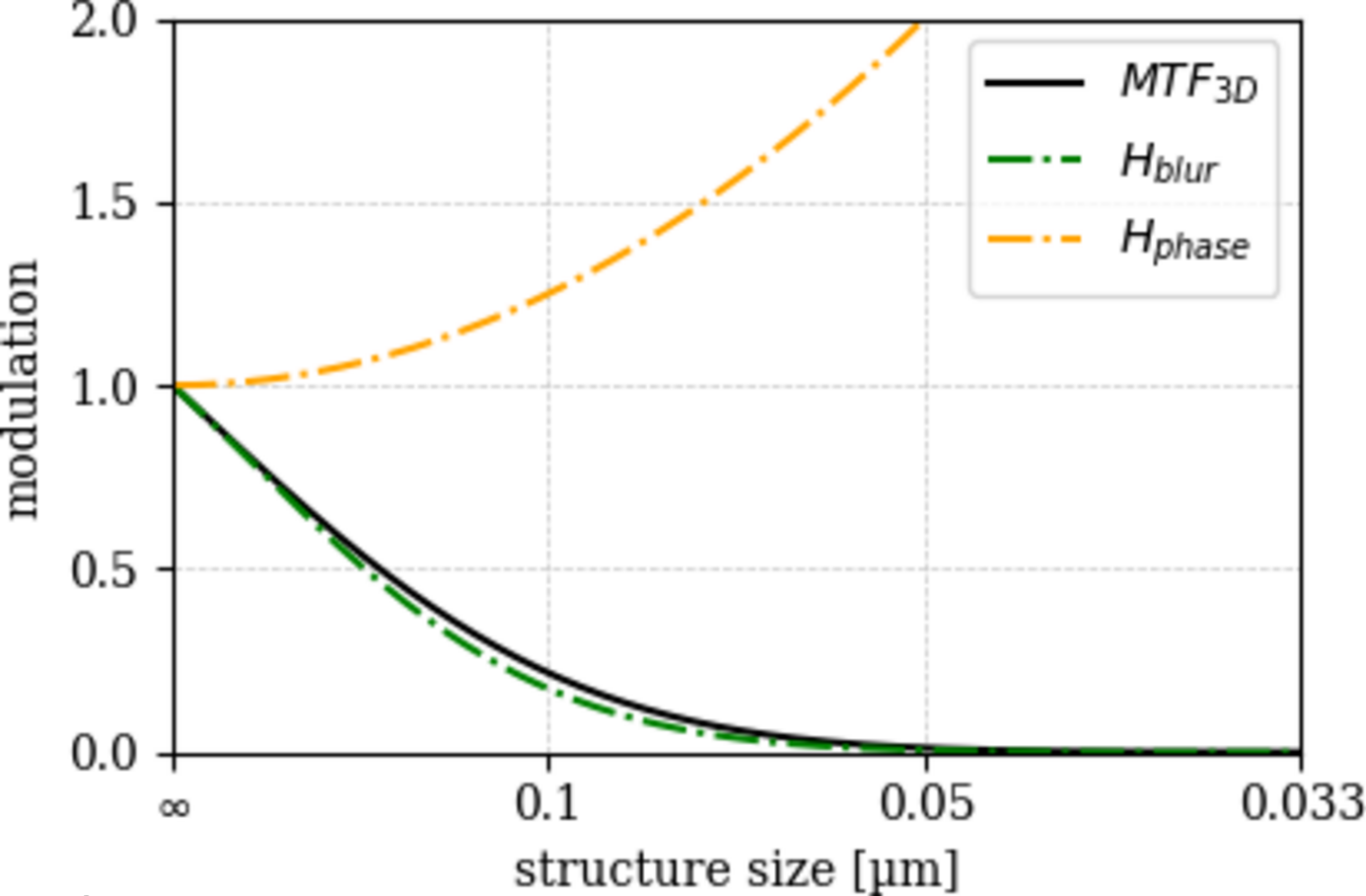
Modulation transfer function (MTF_3D_) and its components: blur transfer function (*H*_blur_) and phase transfer function (*H*_phase_). The shown data are from the ‘esrf: id16b - 29.5kev - holo - 25 nm’ scan.

Modeling the modulation transfer function (MTF_3D_) is achieved by numerically fitting the function *S*_3D_(*u*) (Fig. 2), using the measured radial power spectra shown in Fig. 1 ▸,
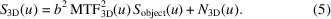
Here, *b* is a scaling parameter, and MTF_3D_(*u*) = *H*_blur_(*u*) × *H*_phase_(*u*) is modeled as the product of two components: a Voigt function *H*_blur_(*u*) describing the blurring effects due to source size and detector resolution (see Fig. 2 ▸), and a phase contrast term *H*_phase_(*u*) = 1 + *p*^2^*u*^2^ which represents propagation-induced edge enhancement. This effect occurs in absorption-mode TXM when slight FZP defocus produces Fresnel fringes that lead to bright rims and the increased brightness of smaller spheres. In Zernike phase contrast, however, the phase ring suppresses such fringes, so no propagation-based edge enhancement is expected.

The estimation of model parameters characterizing the MTF was formulated as the minimization of a composite objective function. The modeled 3D power spectra is denoted by 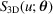, where *u* represents the radial spatial frequency and 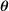 is the set of parameters including those defining the Voigt blur profile (*e.g.* Gaussian and Lorentzian widths) and a phase contrast coefficient governing frequency-dependent edge enhancement. The objective function is defined as a weighted sum of three components: a data fidelity term that enforces agreement between the measured and modeled spectra, a noise penalty term that discourages overestimation of noise power, and a regularization term that constrains the MTF to behave consistently at the spectral boundaries. The total loss is given by
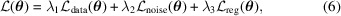
where 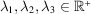 are scalar weights determining the contribution of each term. The data fidelity term is defined as 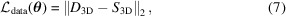
which penalizes deviations between the modeled and empirical spectra. The noise penalty term is expressed as 
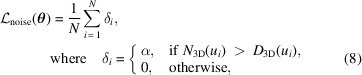

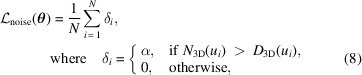
and serves to limit modeled noise that exceeds the measured spectrum in noise-dominated regions. The regularization term constrains the behavior of the MTF at low and high frequencies and is given by 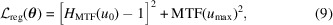
where *u*_0_ and 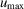 represent the minimum and maximum frequencies, respectively. Optimization of the loss function was carried out using the Covariance Matrix Adaptation Evolution Strategy (CMA-ES), as implemented in the evosax library (Lange, 2022 ▸). CMA-ES is a derivative-free, stochastic optimization algorithm designed for non-convex, multimodal objective landscapes. It proceeds by iteratively sampling candidate solutions from a multivariate Gaussian distribution, updating its mean and covariance based on the fitness of the current population. This approach enables robust estimation of the parameter vector 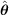 that minimizes 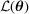, yielding an optimized model for the system’s modulation transfer behavior that adheres to physical and empirical constraints.

### 2.5. Volume image processing

The determination of sphere center coordinates required a multi-step image processing workflow, each step addressing specific challenges in identifying and separating spherical structures.

First, voxel intensities within the sphere material were homogenized using standard filtering techniques, such as Gaussian smoothing, to suppress noise and minimize local intensity variations. This preprocessing improved material uniformity and facilitated reliable segmentation. The specific filter parameters applied are summarized in Table 5 in Appendix *B*[App appb].

Next, the sphere material was segmented from the surrounding background using adaptive, locally optimized thresholding methods. Such approaches are essential for heterogeneous datasets, as they adjust dynamically to local intensity differences and ensure robust isolation of the sphere material as a distinct foreground region.

In cases where adjacent or overlapping spheres were present, a marker-controlled watershed algorithm was employed to separate them. By combining intensity gradients with predefined structural markers, this method reliably delineated individual spheres, even in densely packed regions.

**Figure 3:**
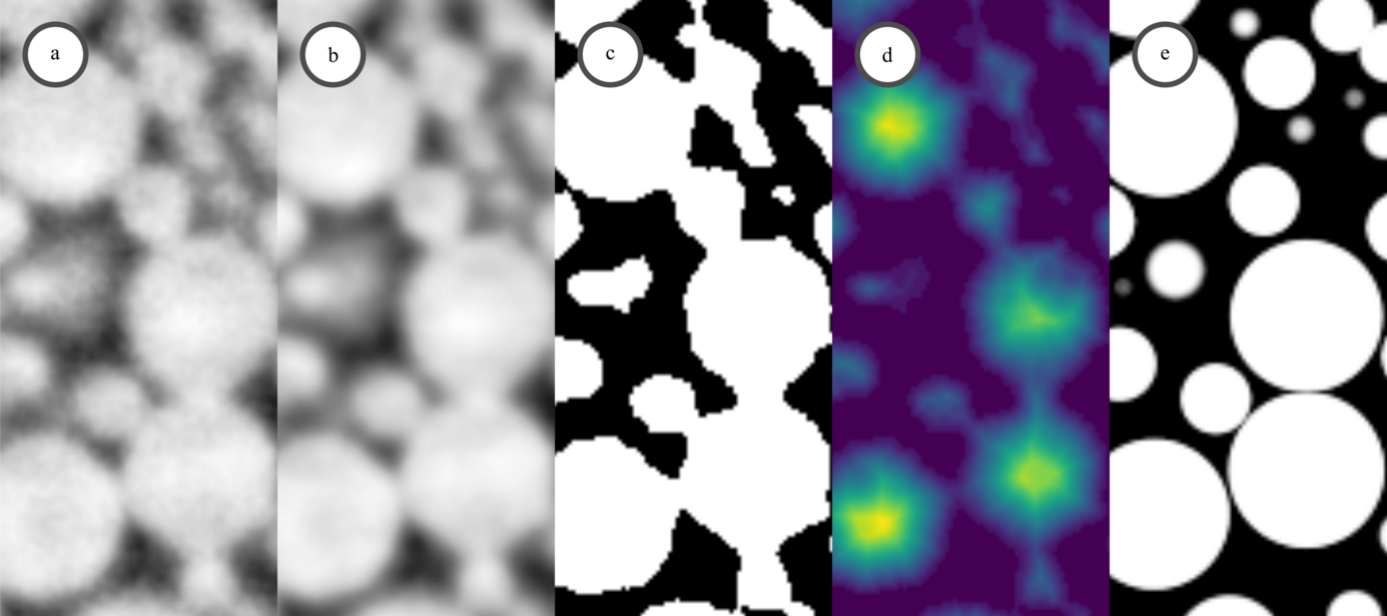
Step-by-step workflow for detecting sphere centers and simulate an ideal scan. (*a*) Raw input image showing densely packed spherical structures with inhomogeneous intensity. (*b*) Gaussian-filtered image for intensity homogenization and noise reduction. (*c*) Binary mask obtained through adaptive thresholding to segment spheres from the background. (*d*) Euclidean distance transform of the segmented regions, highlighting maximal distances from boundaries. (*e*) Final simulated spheres reconstructed supersampled with factor of eight from detected centers and radii.

Following segmentation, a Euclidean distance transform was applied to the binary volume. The resulting scalar field encodes the distance of each voxel to the nearest background voxel. Local maxima within this distance map correspond to the centers of the spheres and were used to extract sphere coordinates with high precision, as illustrated in Fig. 3 ▸.

**Figure 4:**
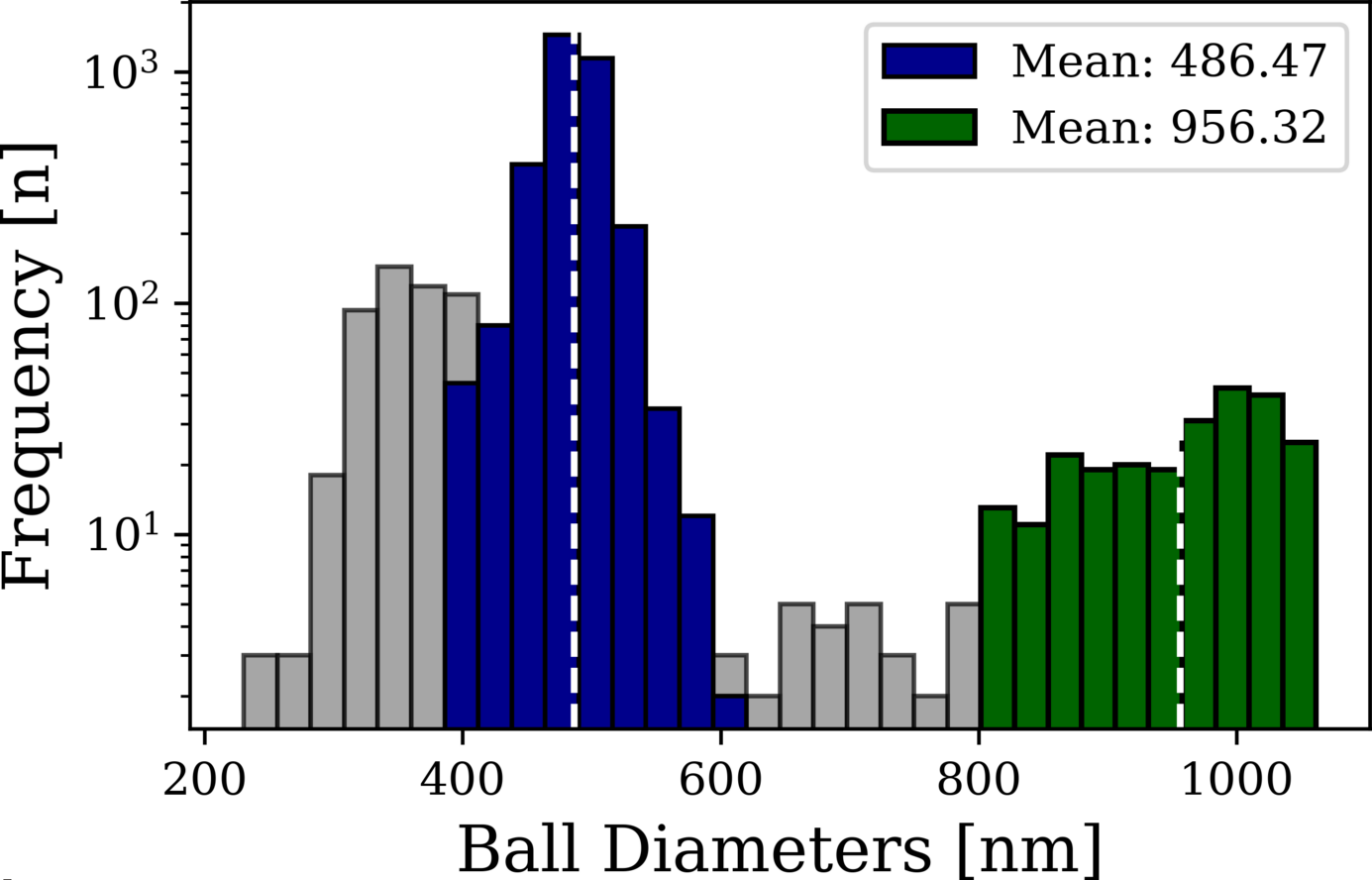
Histogram showing the frequency distribution of the detected spheres following Euclidean distance transform-based center localization from the ‘esrf: id16b - 29.5keV - holo - 25 nm’ scan. The distribution provides quantitative insight into the spatial occurrence and relative size characteristics of the segmented spherical structures within the dataset. Gray shaded bins represent detected spheres or structures that were identified but excluded from further evaluation.

Finally, the distance transform also enabled the estimation of individual sphere radii at their centers. The distribution of detected spheres, including size statistics, is illustrated in Fig. 4 ▸.

Via the distance transform, the radius of each sphere at its center location is also estimated. Using the estimated radii and the known distribution of the set of spheres, the voxel size is approximated as an initial step. Subsequently, an ideal volume is simulated by placing spheres at the identified positions, using the calculated radii to accurately reflect the sphere dimensions. To ensure realistic gray values in the simulated volume, the spheres are supersampled with a factor of eight. This supersampling technique enhances the representation quality by generating higher-resolution data that better approximates the true intensity distribution within the sphere regions.

In a subsequent step, the radial power spectrum of both the original scan and the simulated model is calculated. The spherical arrangement produces a distinctive power spectrum characterized by peaks that correspond to the uniform distribution of spheres. These peaks are a direct result of the structured spatial frequency components inherent to the set of spheres. To refine the voxel size estimation, the peaks are fitted with a linear function. This fitting process is crucial as it significantly improves the accuracy of voxel size determination, which in turn enhances the precision of the MTF estimation. Accurately determining the voxel size is essential for achieving optimal fitting results during the MTF analysis, as any discrepancies in voxel size directly affect the frequency domain representation and subsequent image quality metrics.

## 3. Development of an 3D X-ray phantom for nano-CT

### 3.1. Requirements

For evaluating and comparing instrument performance with respect to general image quality this study follows the blueprint of Zabler *et al.* (2020 ▸). 3D SNR^2^ power spectra as well as MTF_3D_ are evaluated from volume images of randomly clustered spherical particles of two distinct diameters. While our previous study used small capillaries filled with powder (PMMA spheres with diameters of 10 µm and 20 µm were mixed), such a material is not available for much smaller diameters. Sub-micrometre scans were sampling the spheres with 0.5–1.2 µm pixel^−1^ while the present nano-CT scans feature 15–56 nm pixel^−1^, requiring downscaling object and sphere diameters by at least a factor of 20. The following section outlines the manufacturing route for rigid test phantoms whose structure is made of solid spheres of 0.5 µm and 1 µm diameter.

The two diameters should be mixed with identical weight fractions, hence their number repartition should be 1:8 with eight times more small spheres. In contrast to mono-modal clusters, mixed sizes do not display long range ordering. Meanwhile typical nano-CT measurement volumes allow for analyzing 1000–10000 spheres thus guaranteeing homogeneity of the data and its structural content.

With a total field of view of 20–30 µm in diameter, manually cutting and positioning the phantoms is not an option. Furthermore the spheres must form a rigid solid structure that stands free, without a container. For the range of X-ray energies in this study, PMMA shows too little X-ray contrast (absorption or phase contrast). Instead, using silica (amorphous SiO_2_) as material is a much better choice.

Fortunately, silica particles of ideal spherical shape and homogeneous size distribution are largely available in the diameter range from 50 nm to 2000 nm. However, to avoid clotting, which would naturally occur when powders of that sort are kept in ambient air, silica particles are only available as liquid suspensions with a relatively low solid content. Consequently, one must first filter and then bake particle clusters from a large volume of liquid to obtain solid bulk material for further processing into nano-CT phantoms.

The authors acquired two types of dissolved monodisperse SiO_2_-spheres with diameters of 500 mm and 1000 nm from the company EPRUI Biotech, China. According to the manufacturer the standard size deviation for each type should be smaller than 3.5%.

### 3.2. Processing liquid suspensions

Amorphous SiO_2_-spheres suspensions with diameters of 1 µm and 500 nm were used as starting material. First the 20 *w*/*v* aqueous suspensions needed to be vigorously stirred to avoid sedimentation. Secondly, a binder (polyvinyl alcohol with 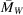 of 31000) was pre-dissolved in distilled water to yield a 3 wt% solution. Then 20 g of each SiO_2_-suspension was mixed with the binder solution to yield a minimum of 0.2 wt% binder with respect to the solid particles weight. The resulting suspension was again stirred and used over several weeks.

**Figure 5:**
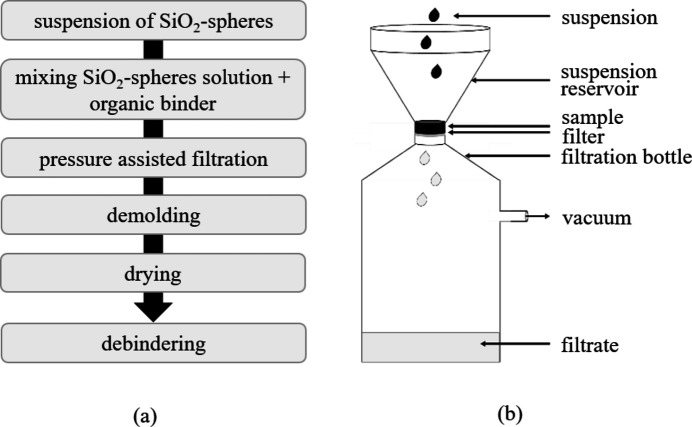
(*a*) Schematic process of sample manufacturing. (*b*) Scheme of pressure filtration.

As a shaping technique, pressure filtration with a polycarbonate membrane with a pore size of 0.4 µm was performed as shown in Figs. 5 ▸(*a*) and 5 ▸(*b*). About 2 mm thick suspension was added at once on top of the filter and, due to vacuum assisted filtration, a cylindrical sample was formed. After about 3 to 5 min the sample was demolded and dried in air for several days.

**Figure 6:**
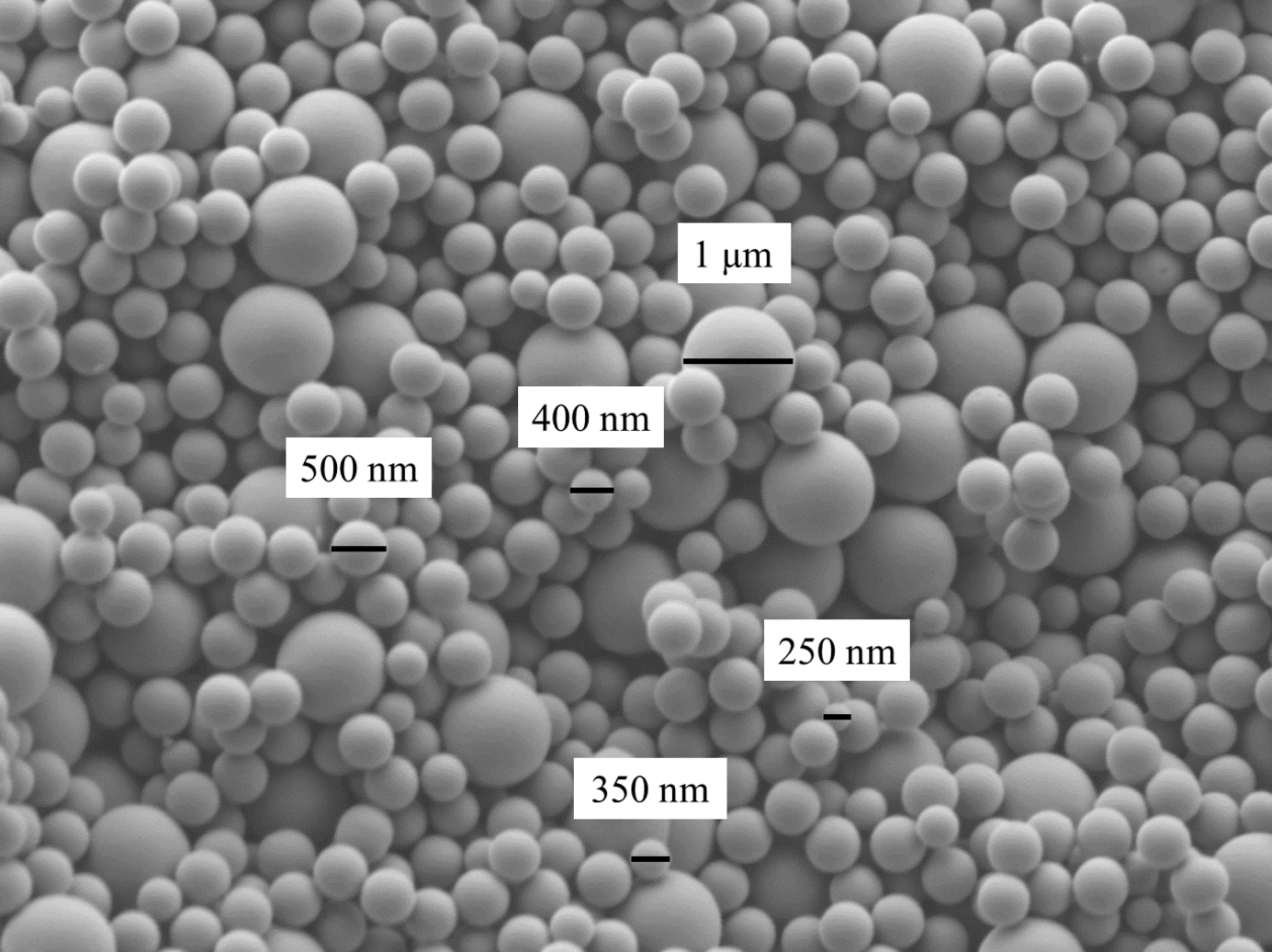
SEM-images of SiO_2_-sphere-samples after debindering at 800°C showing perfect spherical shape and bimodal distribution of particles.

Dry samples with a size of 10 mm in diameter and 1 to 2 mm thickness were debinded, first to burn off the organic binder and second to assure first sintering necks. As debindering temperatures of 650, 700 and 800°C were used. Evaluation using SEM imaging reveal that debindering temperature of 800°C leads to perfect results as presented in Fig. 6 ▸. Sintering necks hold SiO_2_-spheres in place while sphere geometry is still maintained. The 1 µm spheres show perfect accuracy in size, while the batch with 500 nm spheres reveals sphere sizes between 250 and 500 µm.

### 3.3. Sample preparation

**Figure 7:**
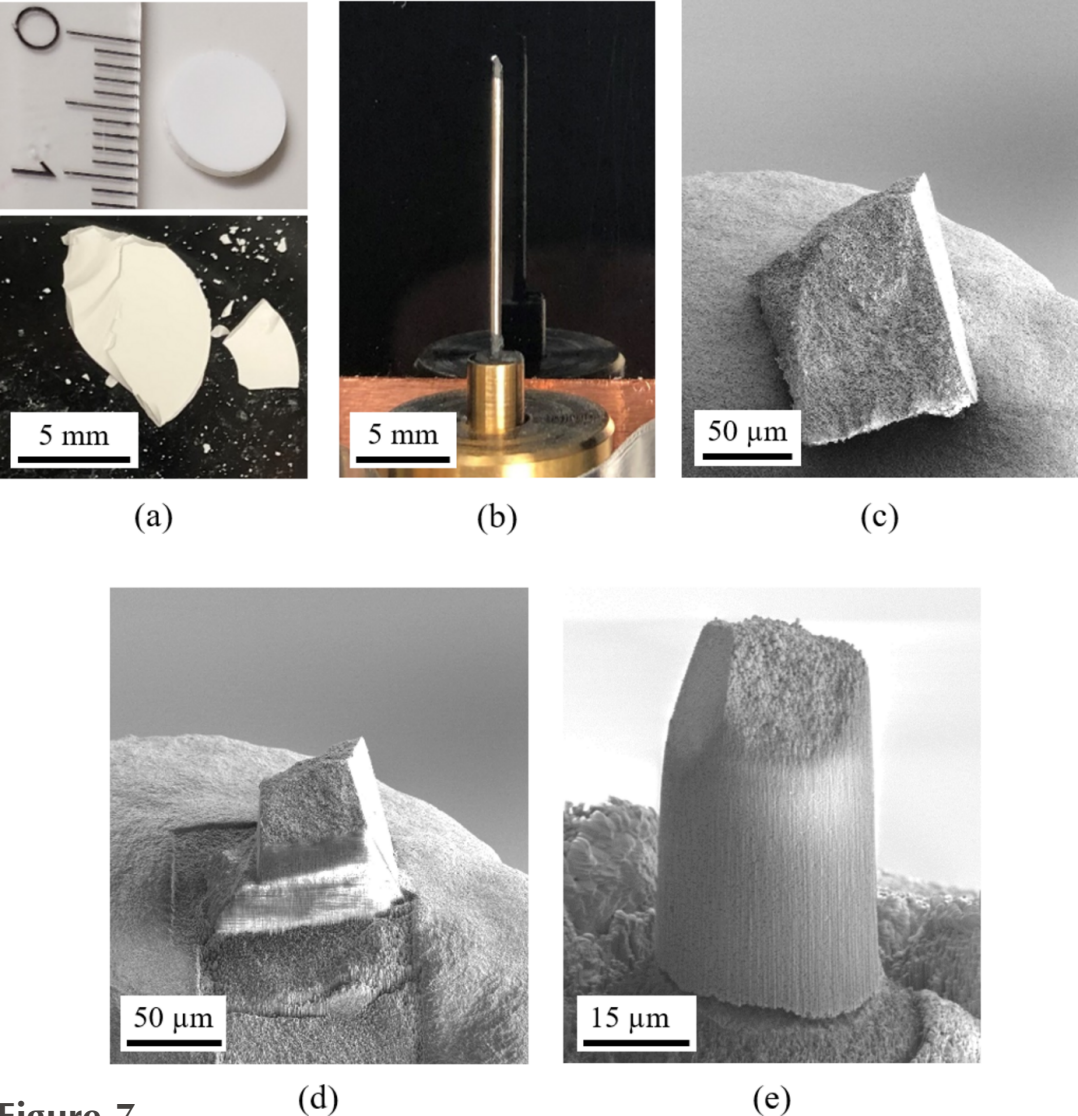
(*a*) SiO_2_-sphere-samples after debindering broken into fragments. (*b*) Fragment glued on pin tip with conductive carbon. (*c*) SEM-image of fragment sputter-coated with 30 nm carbon. (*d*) FIB-preparation showing ion beam removal. (*e*) Prepared ROI in shape of a cylinder with 30 µm in diameter.

To prepare a phantom for nano-CT with a suitable geometry, a dry sample of 10 mm diameter is first broken into small fragments [see Fig. 7 ▸(*a*)]. One of these fragments is then carefully attached to a freely selected pin tip using conductive carbon glue, which is suitable for SEM applications, as shown in Fig. 7 ▸(*b*).

Since electrical conductivity is required for the subsequent focused ion beam (FIB) preparation, the object’s surface is coated with a 30 nm carbon layer using sputter coating. Fig. 7 ▸(*c*) shows the carbon-sputtered fragment resting on the carbon glue imaged by SEM. As illustrated in Fig. 7 ▸(*d*), the fragment is then precisely shaped into a cylindrical form with a diameter of 30 µm using FIB processing (FIB/SEM, Thermo Fisher Helios G4 PFIB CXe, Thermo Fisher Scientific, Waltham, USA). The final geometry of the X-ray phantom is shown in Fig. 7 ▸(*e*). If necessary, a flat surface is prepared to ensure optimal imaging conditions.

Unlike gallium ion implantation, which can alter the material’s properties, the use of a xenon plasma-focused ion beam (Xe-PFIB) does not affect the phantom’s X-ray absorption characteristics in later imaging steps. Additionally, any potential geometric distortions of the spheres due to heat generation during FIB processing can be neglected, as only the region of interest (ROI) at the phantom’s center is utilized for evaluation.

## 4. Round-robin test

**Figure 8:**
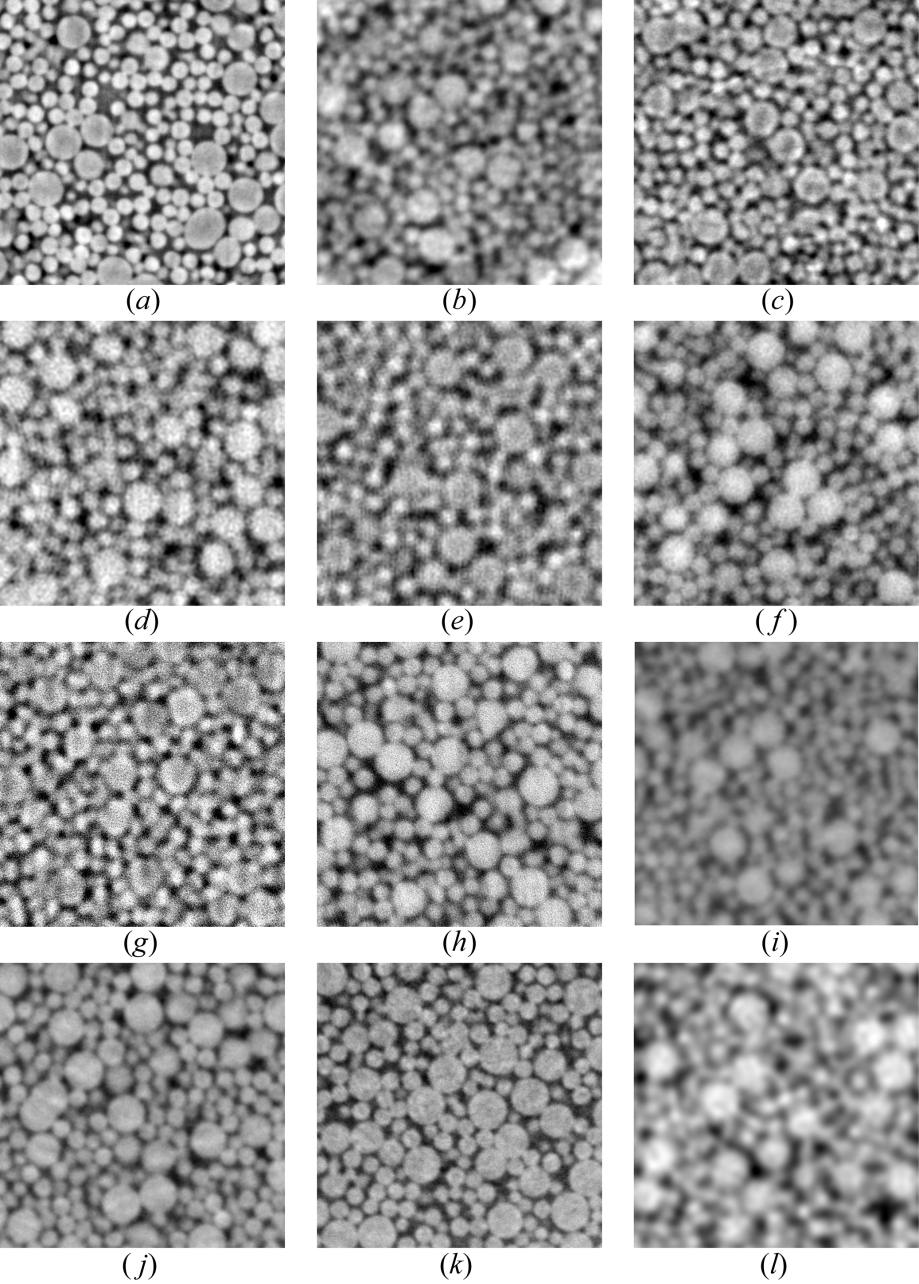
Reconstructed cross-sections of the X-ray phantom: (*a*) Zeiss, (*b*) XRM Pt-BT, (*c*) XRM W-NT, (*d*) SPring-8 micro, (*e*) SPring-8 nano ab 53 nm, (*f*) SPring-8 nano ph 52 nm, (*g*) SPring-8 nano ab 22.5 nm, (*h*) SPring-8 nano ph 18 nm, (*i*) ESRF 50 nm, (*j*) ESRF 25 nm (29.5 keV), (*k*) ESRF 15 nm, (*l*) DESY. The edge length of all images is 8 µm, and the scan parameters correspond to those in Table 2 ▸.

A comparative overview of reconstructed axial (*x*, *y*) slice sections from the evaluated scans and beamlines is displayed in Fig. 8 ▸. While big and small spheres were resolved in all scans, the latter display visible differences in terms of image quality, notably spatial resolution, signal-to noise ratio and image contrast.

Among the laboratory nano-CT scans, the Zeiss X-radia microscope clearly yields the better resolution. At the same time the edges of the bigger spheres display a distinct residual phase contrast, *i.e.* edge enhancement with the spheres’ center visibly darker than their rim. The smaller spheres appear generally brighter even though they are made of the same material. The two scans from the XRM-II microscope are very much different. Using the Pt-bulk target causes stronger image blur whereas the W-needle nano-target yields an image which is visibly sharper. Similar to Zeiss X-radia, the XRM-II W-NT scan displays residual phase contrast, most visibly for the bigger spheres.

The five scans from SPring-8 reveal similar differences. The 38 nm scan from beamline BL20XU appears as equally well resolved as the XRM-II W-NT scan but without edge-enhancement. The scans from beamline BL47XU were recorded at 52 nm or at 18 nm sampling; both recordings were done with and without Zernike phase contrast (labeled ‘pha’ and ‘abs’).

The ‘abs’ scan with 18 nm sampling appears to be slightly better resolved compared with the 52 nm ‘abs’ scan. This observation is less certain for the two ‘pha’ scans. All scans from SPring-8 display relatively strong white pixel noise.

Scans from the European synchrotron (beamline ID16B) are compared at 50 nm, 25 nm and 15 nm sampling at 29.6 keV photon energy. Note that another 25 nm scan was evaluated for *E* = 17.5 keV which is not shown. The 50 nm scan is visibly more blurred compared with the 25 nm scan, while the 15 nm clearly features the sharpest details. No scan from ID16B displays residual edge enhancements. The 56.2 nm scan from PETRA III (beamline P05) appears to be the least sharp with the small spheres barely visible and no residual edge enhancement.

For putting these observations into more quantitative terms, Fig. 13 shows radial intensity profiles from the rim to the center of the small and big spheres, as well as the scattering (STD) of these intensities. The latter were averaged by using the Euclidean distance (EDT) map to create 3D masks and applying them to the volume intensities of all pixels with a given distance from the background. The average intensity of the latter was set to zero and the condition that small and big spheres must have the same background STD in one scan was enforced. Note that these choices leave the absolute intensity scaling of the spheres material open and do not necessarily result in identical intensities for small and big spheres.

Differences in contrast between small and big spheres is observed in the Zeiss X-radia, the XRM-II W-NT as well as the BL47XU ‘abs’ scans (18 nm and 52 nm) with small spheres appearing relatively brighter than the big spheres while the latter show clear edge enhancement (intensity maximum) at the material’s border. The scans XRM-II Pt-BT, BL20XU and ID16B-25nm hardly show any edge-enhancement. These scans’ intensity profiles reach similar values at the big and small spheres center while the image blur (slope at the sphere’s border) does not differ much. The scans BL47XU ‘pha’ (52 nm and 18 nm) as well as the scan from PETRA III and ID16B-50nm show the big spheres in brighter intensity compared with the smaller spheres. The two remaining scans by ESRF-ID16B, *i.e.* 25 nm and 15 nm, show no edge enhancement. Intensity profiles of both scans reach similar values at the spheres’ centers. Note that STD is generally smaller inside the material compared with the air outside. This observation is consistent with SNR depending on material contrast.

Notably, the results and image quality obtained from the laboratory-based X-ray systems are comparable with those from the synchrotron. However, due to the significantly lower photon intensity in laboratory setups compared with synchrotrons, measurement times vary drastically. While measurements in laboratory-based systems take 1 to 2 days, the same measurements at a synchrotron can be completed within 5 to 30 minutes.

To clarify differences between the datasets, we note that the tomographic reconstruction algorithms were not identical across all systems. The Zeiss Xradia 810 Ultra employs a proprietary reconstruction pipeline whose internal implementation is not disclosed by the manufacturer. The ProCon XRM-II nanoCT uses SART with incremental self-alignment and regularization options, while (to the best of our knowledge) all synchrotron datasets (SPring-8, ESRF ID16B and DESY P05) were reconstructed with facility-specific filtered back-projection schemes, with phase retrieval applied beforehand in the case of Zernike or holotomography acquisitions. Drift compensation was applied in all cases.

### 4.1. SNR_3D_ power spectra

**Figure 9:**
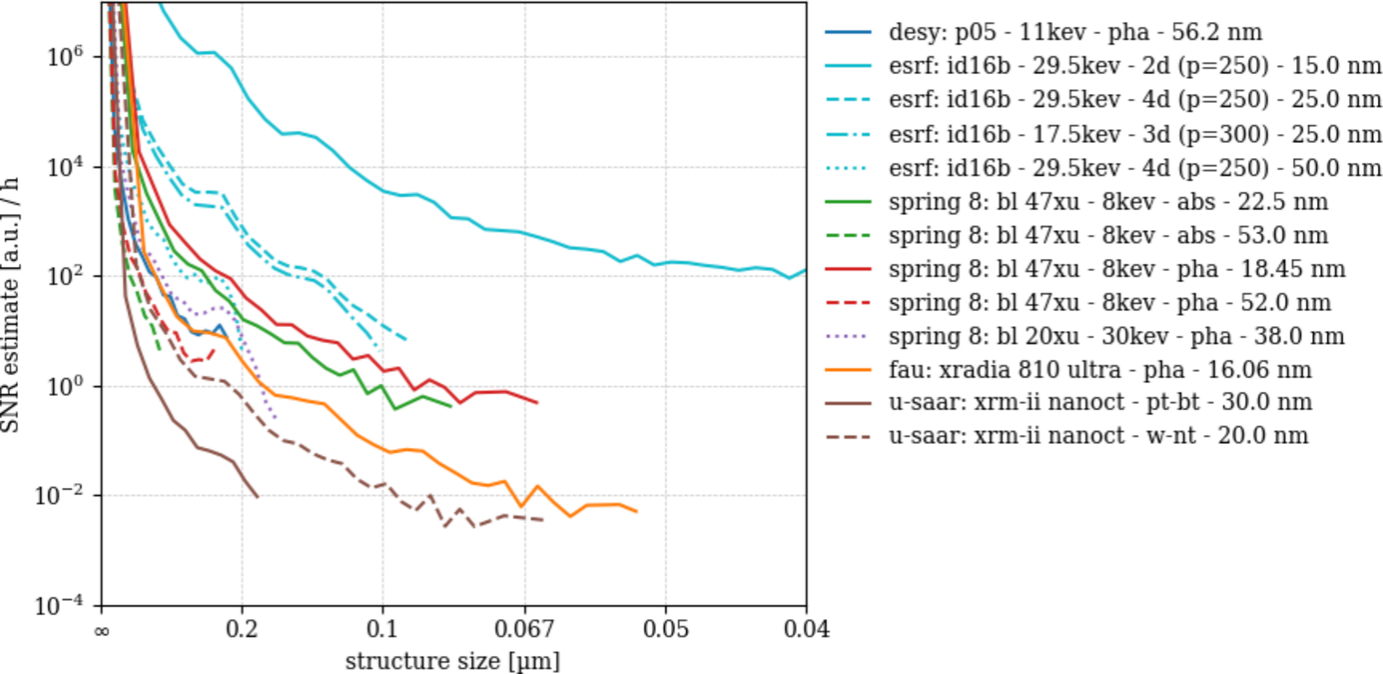
Radial 3D signal-to-noise ratio (SNR_3D_) power spectra of nano-CT scans acquired from various laboratory-based and synchrotron-based systems.

Fig. 9 ▸ presents the radial 3D signal-to-noise ratio (SNR_3D_) spectra for a selection of synchrotron- and laboratory-based nano-CT scans, computed from equally sizes volume image patches [(9 µm)^3^] of the silica sphere phantom.

According to the workflow presented in Section 2.5, spectral signal power is approximated by an ideal noiseless image obtained by structural material segmentation and multiplied by an estimation of the scan’s modulation transfer [*cf*. equation (5)], while noise power is estimated through numerically fitting another model function. The resulting ratio SNR_3D_ therefore approximates the ability of the systems to distinguish the signal corresponding to a certain structural size δ*x* [hence spatial frequency 1/(2δ*x*)] from background noise. SNR_3D_ is normalized with respect to total exposure time during the scans, hence the units of SNR_3D_ (h^−1^).

Comparing the scans’ SNR_3D_ amplitudes at δ*x* = 250 nm (first structural peak of the small spheres) reveals that the former differs by seven orders of magnitude. While the scan ‘esrf: id16b - 29.6kev - holo - 15.0 nm’ features an SNR_3D_(250 nm) amplitude of 862574 h^−1^, ‘u-saar: xrm-ii nanoct - pt-bt - 30.0 nm’ features 0.0916, hence 9.4 million times less. Here, a factor of 2000 is due to the very different scan times while an additional factor of 4700 may be attributed to image quality. Note that our human vision does not perceive image quality as a linear measure; instead it produces a rather binary impression, *i.e.* ‘good’ versus ‘bad’ image quality. SNR_3D_ amplitudes of all scans at δ*x* = 250 nm and 125 nm (if resolved) are listed in Table 3 ▸.

Comparing SNR_3D_ for the two scans by ‘u-saar: xrm-ii nanoct’ reveals that the tungsten nano-target delivers 18 times higher SNR_3D_(250 nm) compared with the bulk platinum target. Comparing the former to the laboratory nano-CT scan ‘fau: xradia 810 ultra - pha - 16.06 nm’ shows the latter featuring seven times higher SNR amplitudes than ‘u-saar: xrm-ii nanoct - w-nt’ and 130 times better than ‘u-saar: xrm-ii nanoct - pt-bt’. Its SNR_3D_(250 nm) value (12) is even higher than one synchrotron scan, namely ‘spring 8: bl 47xu - 8kev - pha - 52.0 nm’ which features 2.83.

Among the four ‘spring 8: bl 47xu’ scans, those with 18.45 nm sampling feature a much higher SNR_3D_(250 nm) compared with the scans with 52 nm sampling: 494 versus 16 for ‘abs’ and 197 versus 2.8 for ‘pha’, respectively. The 38 nm scan from the BL20XU features the intermediate value 33.7. ‘desy: p05 - 11kev - pha - 56.2 nm’, which could be labeled a single-distance holotomography, features SNR_3D_(250 nm) of 23.6.

Scans from the European synchrotron ‘esrf: id16b - 29.6kev - holo’ with 50 nm, 25 nm and 15 nm sampling feature increasingly higher SNR_3D_(250 nm) amplitudes: 159.94, 4161.22 and 862574, respectively. The 25 nm scan was repeated at lower energy (17.5 keV instead of 29.6 keV) which resulted in a lower SNR_3D_: 2468.58.

Like any power spectra, SNR_3D_(*u*) should be an overall monotonously decreasing function, yet all plots in Fig. 9 ▸ start to rise after reaching a minimum value at certain spatial frequencies (*e.g.* ‘esrf: id16b - 29.6kev - holo - 25 nm’ shows this behavior beyond 0.1 µm structure size: it marks the range beyond which SNR_3D_(*u*) cannot be analyzed, the rising amplitude being an artifact of the imperfect noise power fit. We therefore cut off the following DE(*u*) curves at this limit to avoid confusion.

Except for five scans, SNR_3D_ amplitudes could also be evaluated at the second structural maximum of the small spheres (125 nm structure size), showing how steeply SNR_3D_ is decreasing with spatial frequency. At 125 nm the scans ‘esrf: id16b - 29.6kev - holo - 15 nm’ and ‘esrf: id16B - 29.6kev - holo - 25 nm’ show values 34 times and 26 times smaller compared with 250 nm structure size. The amplitude of the ‘fau: xradia 810 ultra - pha - 16.06 nm’ scan is 19 times less at 125 nm. SNR_3D_(*u*) is decreasing even steeper for the 18.45 nm scans from beamline BL47XU at SPring-8: 28 times less for ‘abs’ and 25 times less for ‘pha’. SNR_3D_ amplitudes at 125 nm structure size were also derived for ‘u-saar: xrm-ii nanoct - w-nt - 20.0 nm’ (0.067) and ‘spring 8: bl 20xu - 30kev - pha - 38.0 nm’ (0.012). These values are very low and therefore have to be interpreted with care.

### 4.2. Detection effectiveness DE_3D_

**Figure 10:**
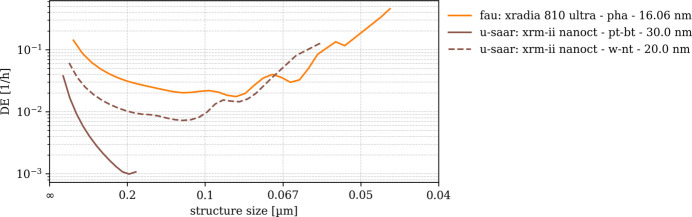
Detection effectiveness (DE_3D_) of laboratory-based nano-CT scans DE_3D_ normalizes the measured signal-to-noise ratio by the ideal structural signal, isolating the imaging system’s intrinsic ability to detect features.

DE is obtained by normalizing SNR_3D_ frequency-wise with respect to the structural amplitudes of S_ideal_. Even though DE is only shown for the frequency range in which we trust SNR_3D_ (*cf*. Fig. 9 ▸) we find DE amplitudes rising or oscillating beyond a certain spatial frequency. Practically, DE(*u*) must be a monotonously decreasing function and therefore only needs to be interpreted to this limit, *e.g.* 0.1 µm structural size for ‘fau: xradia 810 ultra - pha - 16.06 nm’ and 0.125 µm for ‘u-saar: xrm-ii nanoct - w-nt - 20.0 nm’ (*cf*. Fig. 10 ▸). Resulting DE(*u*) spectra are displayed in Figs. 10 ▸ and 11 ▸ for laboratory nano-CT and synchrotron nano-CT separately. The order of the scans and instruments observed from SNR_3D_ amplitudes thereby remains [*e.g.* higher SNR_3D_ shifts the ‘fau: xradia 810 ultra - pha - 16.06 nm’ DE(*u*) values up with respect to ‘u-saar: xrm-ii nanoct - w-nt - 20.0 nm’]. Comparing DE(*u*) for the two ‘u-saar: xrm-ii nanoct’ scans shows that the bulk target (‘pt-bt’) yields a visibly steeper slope due to its worse MTF.

**Figure 11:**
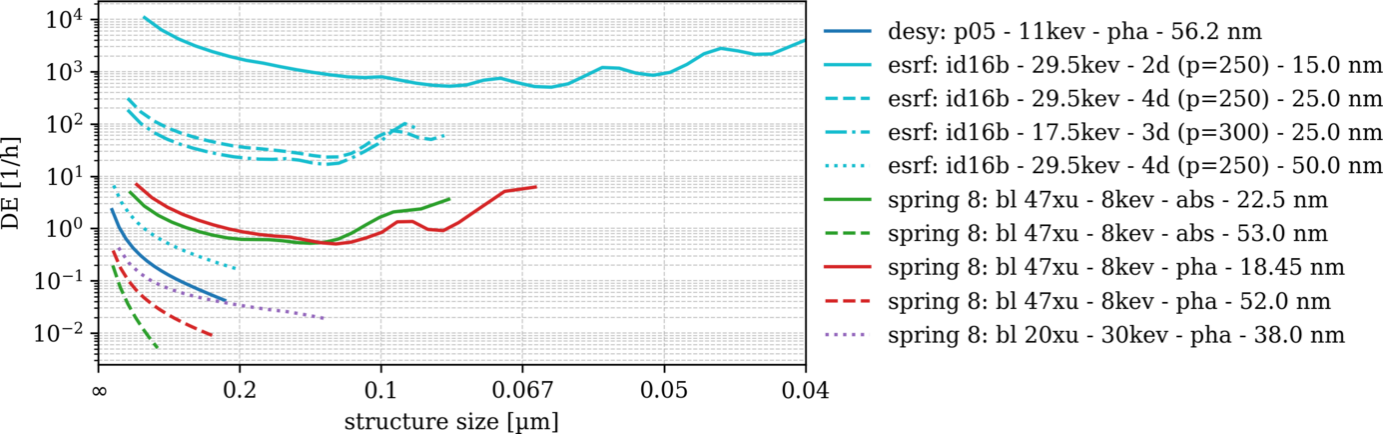
Detection effectiveness (DE_3D_) of synchrotron-based nano-CT scans.

DE(*u*) among the synchrotron scans (Fig. 11 ▸) scales between 10^4^ and 10^−2^ h^−1^. The ‘esrf: id16b’ scans with 15 nm and 25 nm sampling remain in top positions with DE(*u*) of the 15 nm scan almost two orders of magnitude higher than the 25 nm scans. Among the latter, 29.6 keV features higher DE compared with 17.5 keV. The 18.45 nm scans from ‘spring 8: bl 47xu’ feature higher DE(*u*) compared with their 52 nm counterparts, the ‘spring 8: bl 20xu - 30kev - pha - 38.0 nm’ and the ‘desy: p05 - 11kev - pha - 56.2 nm’ scans. Surprisingly, DE(*u*) of the ‘spring 8: bl 47xu - 8kev - abs - 18.45 nm’ scan appears almost horizontal beyond 0.2 nm structure size, making it difficult to determine the limit up to which DE(*u*) can be interpreted before the signal is lost.

### 4.3. Modulation transfer function

**Figure 12:**
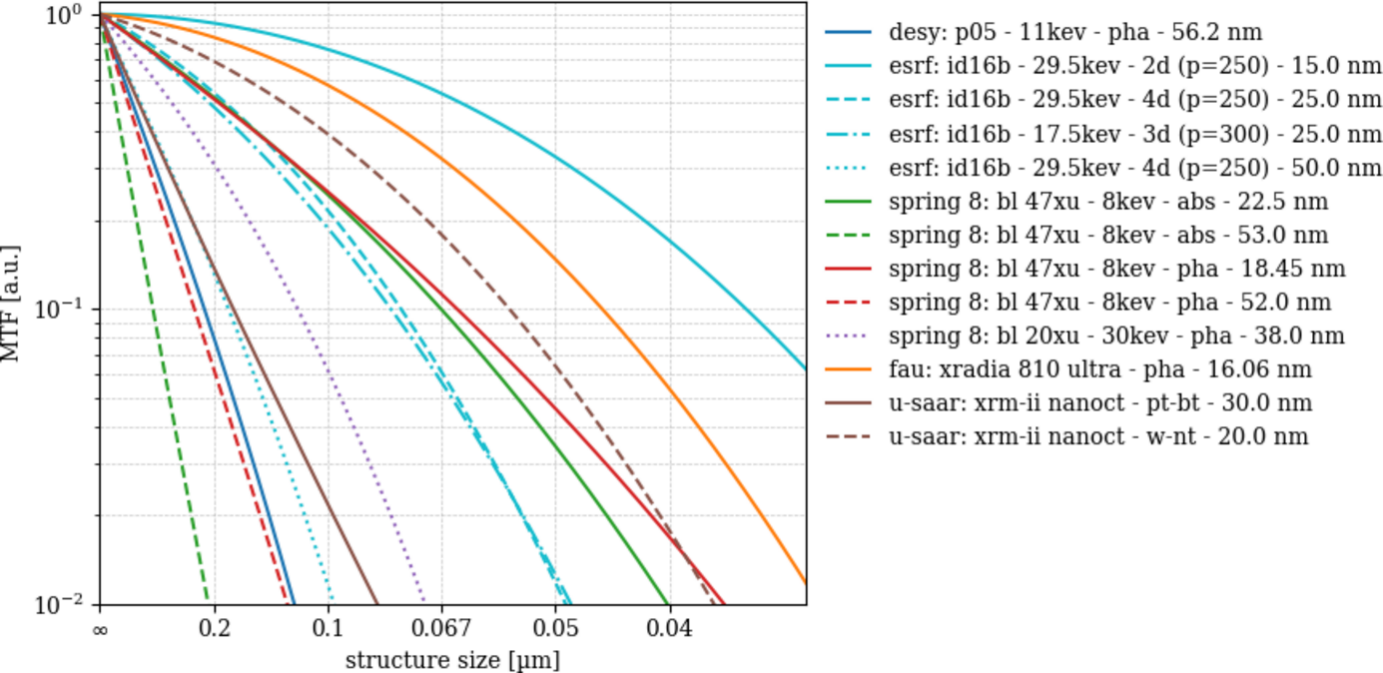
Modulation transfer functions (MTF_3D_) from both laboratory-based and synchrotron-based nano-CT systems.

Fig. 12 ▸ displays the results of numerically fitting the modulation transfer MTF in the measured power spectra to the model [equation (5)] comprising image blur and residual phase contrast. The 10% values of the resulting curves 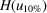 = 0.1 approximate the resolving power of the instruments including image pre-processing, volume image reconstruction as well as post-processing the latter.

There appear to be two batches of scans: one featuring resolving powers below 100 nm, the other above (*cf*. Table 4 ▸). The former comprises ‘esrf: id16b - 29.6kev - holo - 15.0 nm’ (35 nm), ‘esrf: id16b - 29.6kev - holo - 25.0 nm’ (75 nm), ‘esrf: id16b - 17.5kev - holo - 25.0 nm’ (78 nm), ‘spring 8: bl 47xu - 8kev - 18.45 nm’ ‘abs’ (41 nm) and ‘pha’ (64 nm) as well as ‘fau: xradia 810 ultra - pha - 16.06 nm’ (45 nm) and ‘u-saar: xrm-ii nanoct - w-nt - 20.0 nm’ (55 nm).

Among the scans with 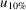 values above 100 nm structure size, two have 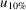 even above 200 nm: ‘desy: p05 - 11kev - pha - 56.2 nm’ (217 nm) and ‘spring 8: bl 47xu - 8kev - pha - 52.0 nm’ (240 nm). The remaining scans feature 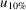 between 100 nm and 200 nm structure sizes. The ‘u-saar: xrm-ii nanoct - pt-bt - 30.0 nm’ features 170 nm compared with 55 nm for the same instrument with tungsten nano-target (w-nt).

### 4.4. Edge spread function

**Figure 13:**
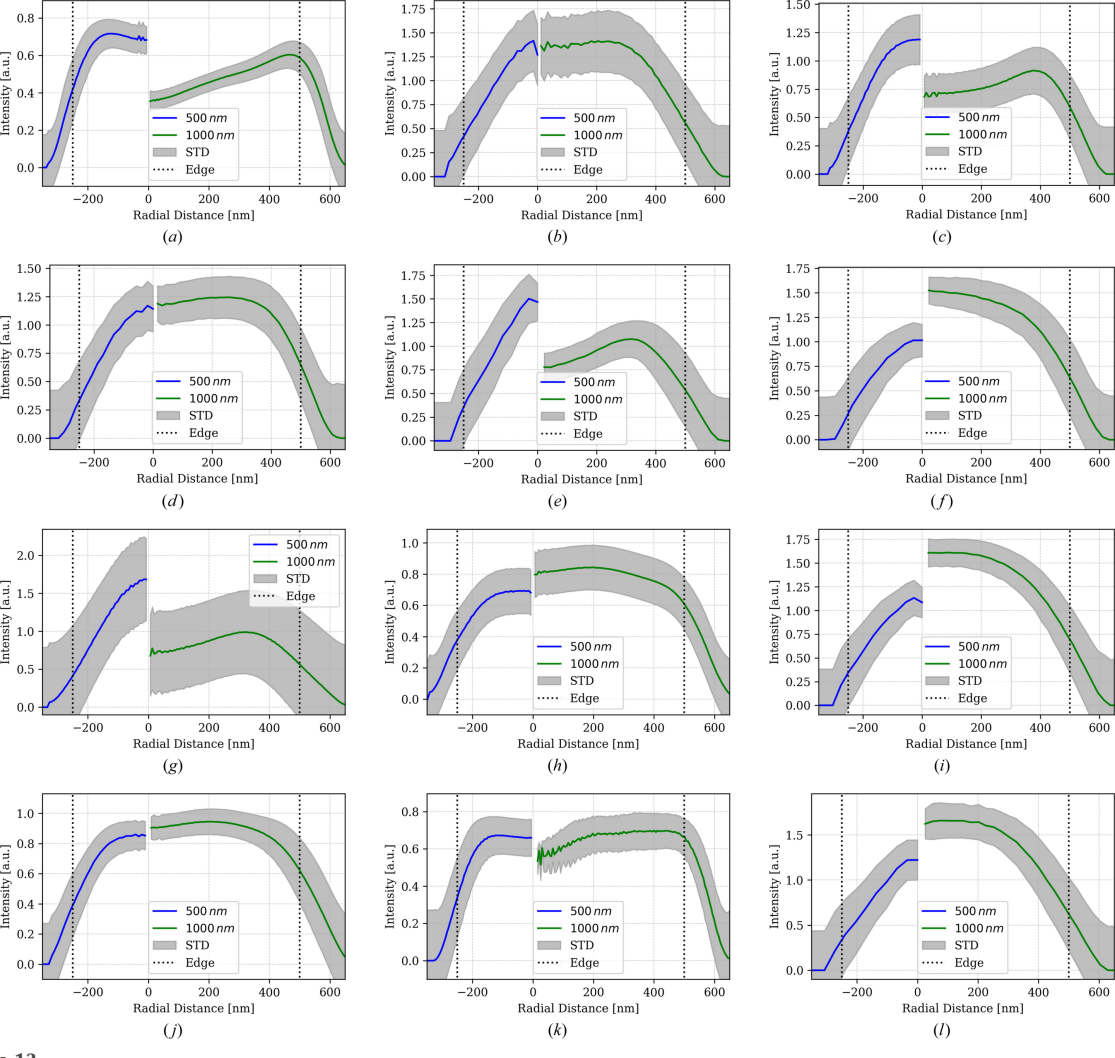
Average radial intensity profiles (calibrated to the mean and STD of air) from center to rim of the small (blue) and large (green) spheres: (*a*) Zeiss, (*b*) XRM Pt-BT, (*c*) XRM W-NT, (*d*) SPring-8 micro, (*e*) SPring-8 nano ab 53 nm, (*f*) SPring-8 nano ph 52 nm, (g) SPring-8 nano ab 22.5 nm, (*h*) SPring-8 nano ph 18 nm, (*i*) ESRF 50 nm, (*j*) ESRF 25 nm, (*k*) ESRF 15 nm, (*l*) DESY. The gray bands mark the intensity scattering. These profiles serve as the basis for the edge spread function analysis described in Section 4.4.

To double-check these estimates of the scans’ resolving power, the edge spread function (ESF; *cf*. Fig. 13 ▸ right-hand side of the intensity profiles) of the larger spheres was also converted into estimates of MTF *u*_10%_ by spatially deriving and Fourier-transforming these edge profiles. The results of this additional resolution estimate are listed in Table 4 ▸ along with the 10% values from the 3D-MTF fit.

Notably, all scans which, according to the latter, resolve structural details finer than 100 nm yield worse resolution when the latter is estimated from ESF: ‘esrf: id16b - 29.6kev - holo - 15.0 nm’ yields 68 nm instead of 35 nm, ‘esrf: id16b - 29.6kev - holo - 25.0 nm’ 165 nm instead of 75 nm, ‘esrf: id16b - 17.5kev - holo - 25.0 nm’: 132 nm instead of 78 nm, ‘fau: xradia 810 ultra - pha - 16.06 nm’ 66 nm instead of 45 nm, ‘u-saar: xrm-ii nanoct - w-nt - 20.0 nm’ 96 nm instead of 55 nm and ‘spring 8: bl 47xu - 8kev - pha - 18.45 nm’ yields 112 nm instead of 64 nm. Most particularly, ‘spring 8: bl 47xu - 8kev - abs - 18.45 nm’, previously the second best resolving scan, according to its ESF, resolves only 171 nm instead of 41 nm, hence four times worse.

Meanwhile, ESF analysis of the scans ‘esrf: id16b - 29.6kev - holo - 50.0 nm’, ‘u-saar: xrm-ii nanoct - pt-bt - 30.0 nm’, ‘spring 8: bl 20xu - 30kev - pha - 38.0 nm’ and ‘spring 8: bl 47xu - 8kev - abs - 52.0 nm’, all of them featuring resolving powers between 115 nm and 170 nm, match the *u*_10%_ values of the 3D-MTF model perfectly well.

For the remaining scans, which previously featured lower resolutions, estimating the latter from the ESF profiles produces even better values compared with *u*_10%_ from 3D-MTF fits. ‘desy: p05 - 11kev - pha - 56.2 nm’ would resolve 166 nm instead of 217 nm, ‘bl47xu 8 pha 52 nm’ achieves an even better score of 137 nm. Note that all scans, according to ESF analysis, resolve details below 200 nm.

## 5. Discussion

This study evaluates spatial resolution as well as SNR_3D_ and DE power spectra with respect to measurement time of a new silica sphere phantom material which was produced uniquely for this purpose. A total of 13 nano-CT scans featuring voxel sizes ranging from 16 nm to 56 nm and photon energies ranging from 5.4 keV to 30 keV were recorded separately on six instruments, four of which were synchrotron beamlines (ESRF, SPring-8 and DESY) and two laboratory nano-CT scanners (Procon XRM-II nano-CT and Zeiss Xradia Ultra 810).

Lower image quality in some datasets was not attributable to mechanical instabilities. To our knowledge, all scans were inspected for drift by analyzing sinogram consistency and, where applicable, cross-correlating the first and last projection of the acquisition. For the synchrotron datasets, the sinograms did not show evidence of systematic lateral shift, wobble or geometric distortion, beyond a linear shift. For the laboratory systems, the ProCon XRM-II nanoCT employs incremental self-alignment within its SART reconstruction, compensating residual sample-stage motion. The Zeiss Xradia 810 Ultra provides an internal projection-alignment option, although the underlying algorithm is proprietary and cannot be evaluated in detail. Since the SPring-8 reconstruction workflow is not fully documented to the user, we cannot assess whether internal alignment steps were applied; however, the sinograms themselves did not indicate measurable drift. The observed variations in image quality therefore arise primarily from differences in photon flux, detector blur, scintillator–optics coupling, contrast mechanism (absorption versus phase), and reconstruction strategies, rather than from mechanical instabilities during scanning.

While these instruments differ very much technically (some even permit scanning with different parameters for each instrument), the resulting volume images could be analyzed by one reproducible and consistent evaluation framework across all scans, enabling a consistent, reproducible workflow for the entire dataset. Sample material is now available in abundant quantity and the data analysis pipeline is published along with this study. Combined, these two guarantee the reproducibility and feasibility of this test to all participants and the scientific community. A dedicated repository was created, including the complete dataset used for analysis^1^. The evaluation process described in this study is implemented through a GitLab CI (continuous integration) pipeline, which automates the analysis and generates the resulting metrics and figures as a structured .h5 output file.

### 5.1. Regarding signal-to-noise ratio

Changing the data acquisition software of the instruments so that it would record and reconstruct two identical scans at the same time (*e.g.* by doubling, then splitting the number of projections in two and reconstructing the corresponding volume images separately) would benefit SNR_3D_ analysis and hence MTF and DE analysis a lot. While this study relies on the quality of fitting numerical model to *N*_3D_ and MTF, having two identical copies of the resulting volume image would allow for analytically computing SNR_3D_ without any need for a model (Ullherr & Zabler, 2019 ▸). However, during this study this was not an option which is why we relied on model functions.

Compared with SNR_3D_, DE eclipses the influence of the sphere phantom’s structure factor while preserving other factors like contrast, photon flux, spatial resolution (MTF), noise power and noise correlation, all of which compose SNR_3D_. The laboratory nano-CT scans show a clear pattern: ‘fau: xradia 810 ultra - pha - 16.06 nm’ shows approximately three times higher DE than ‘u-saar: xrm-ii nanoct - w-nt - 20.0 nm’ with both instruments most likely being at their peak performance. While the first uses FZP to collect and project monochromatic X-rays onto the detector, the second uses a polychromatic cone beam without any optical elements. No such pinhole-type imaging device will ever achieve the light-collection efficiency of a lens-based microscope such as the Xradia 810 Ultra. However, being a relatively simple upgrade option for scanning electron microscopes, the XRM-II nanoCT has its own merits.

SNR_3D_ and DE show alike that, for the ‘u-saar: xrm-ii nanoct’, choosing the shape and material for the X-ray anode is of highest importance. A tungsten nano-target with a tip size of approxately 30 nm yields a ten times better DE at 200 nm structure size, compared with a bulk platinum target. The present results clearly show that the higher electron absorbance of bulk platinum (*i.e.* 30 nm tungsten is partly transparent to 30 keV electrons) does not compensate for the worse MTF, *i.e.* the point spread due to electron diffusion inside the bulk target.

Among the synchrotron sources, the ESRF clearly retains its pioneering role of an ‘extremely bright source’ which it has given itself after completing its upgrade in 2022. At 250 nm structure size, a DE of the ‘esrf: id16b - 29.6kev - holo - 15.0 nm’ scan is 1000 times the DE of ‘spring 8: bl 47xu - 8kev - abs - 18.45 nm’. For coarser spatial sampling (50 nm for esrf: id16b, 52 nm for spring 8: bl 47xu and 56.2 nm for desy: p05) this lead remains with the ESRF ID16B having approximately five times higher DE than its competitors. Note that, despite projecting a cone beam, the ESRF ID16B instrument collects the synchrotron light through highly efficient X-ray optics, namely bent KB-mirrors which are not inferior to FZP in terms of efficiency.

One result which stands out for all synchrotron scans is that making spatial sampling coarser results in drastically worse SNR_3D_ and DE. ‘spring 8: bl 47xu’ were using one FZP for all scans: a 1 µm-thick tantalum FZP with Δ*r* = 50 nm, *r*_*N*_ = 620 µm and *f* = 200 mm at 8 keV X-ray energy. The FZP’s resolving power should be 61 nm at best. According to equation (1), first order diffraction should ideally have 25% efficiency for this lens, independent of the object sampling. The main difference between the 52 nm and the 18.45 nm scans is the scintillator which was coupled through lens-optics to an ORCA Flash4.0 sCMOS camera. For the 18.45 nm, a 100 µm LuAG-screen was coupled through microscope 10× tandem optics yielding a screen sampling of 0.65 µm, while the 52 nm scans used 200 µm LuAG coupled to a tandem of *f* = 20 mm and *f* = 85 mm lenses. These two screens would ideally yield detector resolutions of 2.3 µm and 1.6 µm for the 200 µm and the 100 µm screen, respectively, assuming an optimal numerical lens aperture and perfect chromatic corrections. The 52 nm lens tandem featuring a very low NA would explain the difference in SNR_3D_ and the worse resolution, compared with the 18.45 nm scans.

In contrast, the ‘esrf: id16b’ scans were all recorded with the same detector: a 30 µm thick LSO scintillator which is imaged onto a PCO edge 4.2 sCMOS camera through 10× microscope tandem optics. The resulting images could ideally achieve 0.9 µm constant screen resolution. Propagation-based phase contrast of the ‘esrf: id16b’ scans increases with longer focus–object distance *l*, hence with coarser object sampling since the total focus–detector distance *d* + *l* = 705 mm was constant. The resulting edge enhancement through Fresnel fringes should benefit the overall SNR_3D_ of these scans, but our results indicate the opposite. Closer inspection reveals that the radius of the first Fresnel zone *r*_F_ = (λ*d*_eff_)^1/2^ [where *d*_eff_ = *dl*/(*d* + *l*) is at least ten times bigger than the estimated resolving power of the scans (Cloetens *et al.*, 1999*a* ▸)]. Consequently, propagation cannot enhance contrast further. It is possible that the ‘esrf: id16b’ scans’ differences in terms of SNR_3D_ and DE stem from performance of the iterative phase retrieval process which applies prior to filtered back projection and which may perform better for shorter propagation distances, hence finer object sampling. In that respect, the ‘esrf: id16b - 25.0 nm’ scan recorded at 17.5 keV X-ray energy yielding lesser SNR_3D_ and DE amplitudes compared with the recording at 29.6 keV energy would feature a larger *r*_F_ similarly to a longer propagation distance, and thus suffer from the same effect.

### 5.2. Spatial resolution: MTF and ESF

Regarding the MTF_10%_ values estimated by fitting the scans’ radial power spectra, we observe differences which primarily coincide with the differences in SNR_3D_ and DE: finer object sampling with the ‘esrf: id16b - 29.6kev - holo’ as well as the ‘spring 8: bl 47xu - 8kev’ scans produce sharper resolution. The best records are ‘esrf: id16b - 29.6kev - holo - 15.0 nm’ with a MTF_10%_ value of 35 nm an MTF_ESF,10%_ value of 67 nm as well as ‘fau: xradia 810 ultra - pha - 16.06 nm’ with a MTF_10%_ of 45 nm and an MTF_ESF,10%_ value of 66 nm. The shorter values of MTF_10%_ compared with MTF_ESF,10%_ of these scans likely result from the former’s model which integrates a term for residual phase contrast, thus shifting the resolution limit to higher frequencies. For some of the coarser scans the opposite is observed, *i.e.* MTF_ESF,10%_ are more optimistic than the MTF_10%_ model (*e.g.* ‘spring 8: bl 47xu - 8kev - 52 nm’ and ‘desy: p05 - 11 keV - 56.2 nm’ scans). Such differences are less related to phase contrast in the MTF and more likely to ambiguities of the NPS model fit. Depending on how well the NPS model fits the actual data a more or less optimistic MTF is used for modeling the signal PS. In contrast, noise is removed by averaging the ESF profiles prior to calculating the spatial derivative and Fourier-transform of the latter. However, this spatial averaging might blur the residual phase contrast which is observed in the scans’ sections. The actual resolving power of each instrument lies probably in between the two estimates.

Both absorption-contrasted scans from ‘spring-8: BL47XU’ (22.5 nm and 53.0 nm) strongly differ in 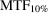 values, depending on whether the latter are obtained though SNR_3D_ or ESF analysis. For the 22.5 nm sampling, NSR_3D_ analyis yield 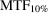 = 66.7 nm while 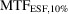 is 162.6 nm, 2.4-times worse. For the coarser 53 nm scan 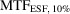 = 123.8 nm while 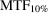 is now almost four times worse (*cf*. Table 4 ▸). These large discrepancies between two analysis methods could stem from a strong uncertainty in the SNR_3D_ analysis which estimates MTF_3D_ by fitting a model function. While ‘spring 8: bl47xu - abs - 53 nm’ only covers a limited spectrum thus making the space of good fitting parameters larger, the ‘spring-8: bl47xu - abs - 22.5 nm’ scan feature a relatively constant SNR_3D_ over a broader frequency band which makes the quality of the MTF_3D_ fit equally uncertain. ESF analysis is likely more robust in these two cases.

The lower resolution of the ‘desy: p05 - 11kev - pha - 56.2 nm’ scan compared with multi-distance holotomography scans by the ESRF is likely not related to the virtual focal spot created by the FZP which should be similar to the focal spot of SPring-8 FZP, *i.e.* 61 nm. Image processing and/or detector blur may further reduce the scan’s spatial resolution, but to be sure future experiments are required.

## 6. Conclusion

Instrument resolution of at least 200 nm, preferably below 100 nm, is required for this test to apply. Judging from edge profiles (Fig. 13 ▸), all reported scans met the first criterion, while 7 out of 13 met the second, comprising laboratory and synchrotron instruments. Note that we did collect scans from nanoCT instruments that could not resolve the phantom structure. For fairness reasons, we only report the successful cases.

All estimated resolution limits stayed below the estimated structural sizes up to which SNR_3D_ and DE of the scans could be analyzed, before their power spectra became pure noise. This observation clearly indicates that all scans would have resolved more details if their total exposure time had been extended. Another consequence is that estimating 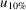 from fitting the MTF model to SNR_3D_ is in most cases an extrapolation. The limited validity of the latter explains some of the differences between the resolution estimates of MTF_3D_ and the ESF analysis. Despite these uncertainties, our results can be compared with resolution estimates from previous studies. Our results roughly confirm the 50 nm resolving power of the Xradia Ultra microscope estimated by Epting *et al.* (2012 ▸). The 35 nm to 67 nm we find for the 15 nm scan from ESRF ID16B beamline are the best 3D-results reported from this beamline so far. Likewise, a recent report using the XRM-II nanoCT, by Lutter *et al.*, reported 150 nm while our test yields 55 nm to 96 nm, hence significantly higher resolving power. Note that the scans from this study were provided by U-Saar which operates a newer version of the XRM-II scanner compared with Lutter *et al.*(2021 ▸). During the evaluation of the data, specifically the attenuation-contrasted (abs) SPring-8 scans, we observed slight discrepancies in the provided voxel sizes. We therefore estimated the effective voxel size directly via the spatial distribution of the detected spheres within the phantom to ensure the accuracy of the resolution metrics. The 176 nm resolution reported by Flenner *et al.* (2020 ▸) who used very much the same ‘nano-holotomography’ experiment for this study are very close to the 166 nm estimated from 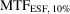 while 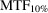 estimates a slightly higher value of 217 nm. Note that better resolving powers are reported from the P05 beamline when the FZP is used in TXM configuration or when finer object sampling is applied (Larsson *et al.*, 2019 ▸). Flenner *et al.* claim that a detector blur of 3 pixels explains these variations.

The SNR_3D_ and DE power spectra provide additional important facts about the scans’ image quality which is influenced by many factors, including modulation transfer. In particular, for SNR_3D_ and DE power spectra obtained from some instruments repeatedly in different configurations (energy, object sampling, phase ring, anode material), results indicate that some instruments harbor substantial room for parameter optimization. Choosing a better set of instrument parameters improves spatial resolution as well as SNR_3D_. The outstanding high SNR_3D_ and DE of the ESRF ID16B 15 nm scan shows how much potential may reside in optimizing these quantities, *e.g.* with respect to propagation, hence *r*_F_, while keeping an eye on the modulation transfer that applies during the iterative phase retrieval process.

Likewise, the screen resolution of any lens-coupled indirect detector (LCID) should be verified with respect to the total resolution of the nanoCT volume image. It is common knowledge that a lack of chromatic lens correction (for the emission wavelength of the scintillator) or a bad match between the screen thickness and the lens aperture causes strong image blur. Low collection and low quantum efficiencies of the LCID may cause avoidable losses in terms of SNR_3D_ power.

Regarding FZP microscopes, we lack a detailed understanding of how exactly using Zernike phase rings influences the spatial resolution of the scans. The latter appear slightly degraded compared with attenuation scans at the SPring-8 beamline BL47UX.

In summary, this test is not an evaluation of the instruments best performances. On the contrary, our results hint that most instruments would benefit from systematically optimizing their scanning parameters. This study is a recipe for obtaining absolute and relative 3D image quality metrics (*i.e.* MTF, SNR_3D_ and DE) for achieving optimal, comparable and reproducible settings for each instrument. Our method should be applied to systematically rule out factors for image quality loss and consequently optimize the instruments performance.

## Figures and Tables

**Figure 14:**
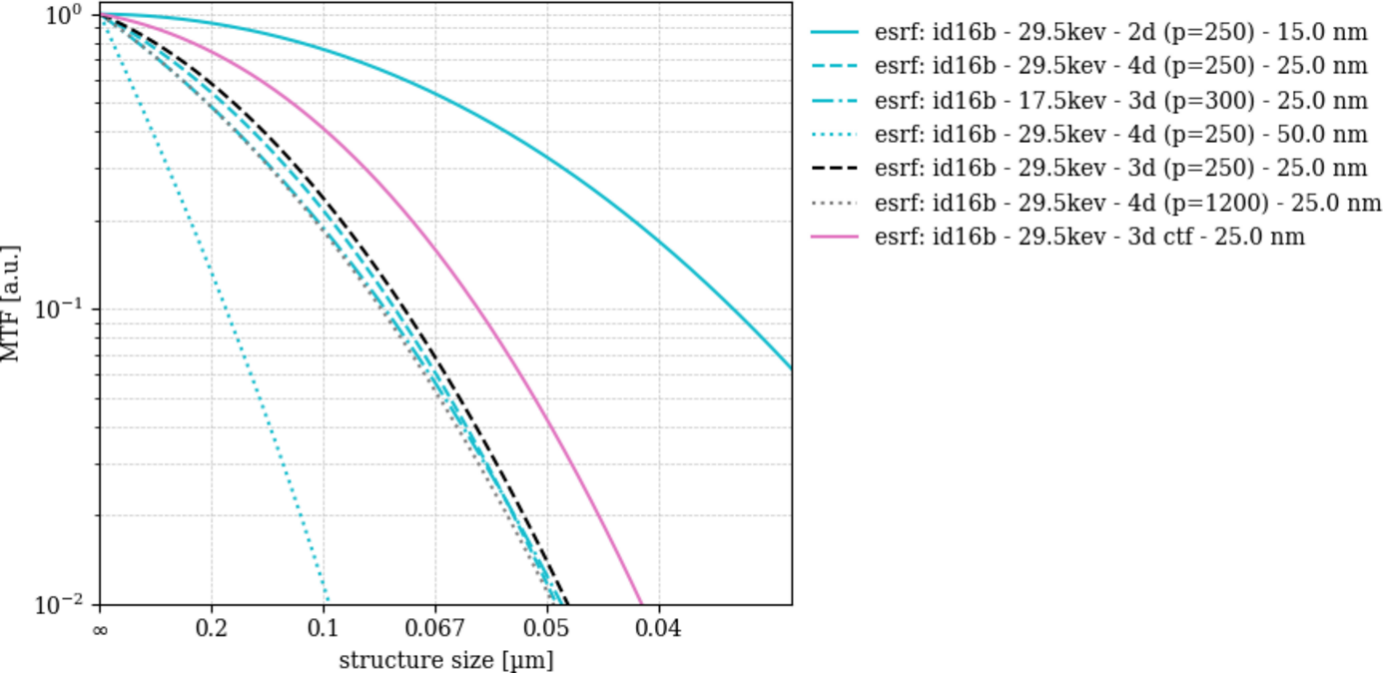
Modulation transfer functions (MTF_3D_) ESRF scans with different algorithm parameters.

**Figure 15:**
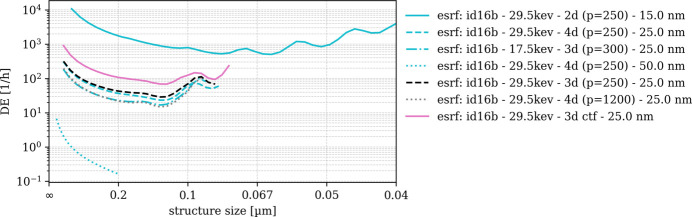
Detection effectiveness (DE_3D_) of ESRF nano-CT scans with different algorithm settings DE_3D_ normalizes the measured signal-to-noise ratio by the ideal structural signal, isolating the imaging system’s intrinsic ability to detect features.

**Table 1:** Participating systems and beamlines

System / beamline	Type	Affiliation and location
Zeiss Xradia 810 Ultra	Lens-based	Micro- and Nanostructure Research, Friedrich-Alexander University, Erlangen, Germany
ProCon XRM-II nano-CT	Source-based	Correlative Microscopy and Tomography, Saarland University, Saarbrücken, Germany
SPring-8 BL20XU	Synchrotron	Japan Synchrotron Radiation Research Institute, Sayo, Hyogo, Japan
SPring-8 BL47XU	Synchrotron	Japan Synchrotron Radiation Research Institute, Sayo, Hyogo, Japan
ID16B	Synchrotron	European Synchrotron Radiation Facility, Grenoble, France
P05 PETRA III	Synchrotron	Deutsches Elektronen-Synchrotron (DESY), Hamburg, Germany

**Table 2:** Equipment and scan parameters of the CT systems and beamlines

Scan acronym	System / beamline	Method / contrast	Hardware	Scan parameters
fau: xradia 810 ultra - pha - 16.06 nm	Zeiss Xradia 810 Ultra	Zernike phase contrast	Phase ring; 1024 × 2024 scintillator	5.4 keV Cr *K*_α_; *t*_exp_ = 200 s; projections = 901; voxel size = 16.06 nm
u-saar: xrm-ii nanoct - pt-bt - 30.0 nm	ProCon XRM-II nanoCT	Transmission X-ray microscopy / absorption	Pt bulk target; ADVACAM WidePIX MPX3 CdTe 55 µm; Kleindiek Nanolathe	30 keV Pt-spectrum; *t*_exp_ = 90 s; projections = 2161; voxel size = 30 nm
u-saar: xrm-ii nanoct - w-nt - 20.0 nm	ProCon XRM-II nanoCT	Transmission X-ray microscopy / absorption	W needle target; ADVACAM WidePIX MPX3 CdTe 55 µm; Kleindiek Nanolathe	30 keV W-spectrum; *t*_exp_ = 90 s; projections = 2161; voxel size = 20 nm
spring 8: bl 20xu - 30kev - pha - 38.0 nm	SPring-8 BL20XU	Zernike phase contrast	Phase ring; BeamMonitor (*f* = 105 mm, LuAG 200 µm); Hamamatsu Orca-Flash 4.0 CMOS 6.5 µm	30 keV monochromatic; *t*_exp_ = 500 ms; projections = 1800; voxel size = 38 nm
spring 8: bl 47xu - 8kev - abs - 53.0 nm	SPring-8 BL47XU	Absorption contrast	BeamMonitor2 (*f* = 20 mm, LuAG 200 µm); Hamamatsu Orca-Flash 4.0 CMOS 6.5 µm	8 keV monochromatic; *t*_exp_ = 200 ms; projections = 5000; voxel size = 52 nm
spring 8: bl 47xu - 8kev - pha - 52.0 nm	SPring-8 BL47XU	Zernike phase contrast	Phase ring; BeamMonitor2 (*f* = 20 mm, LuAG 200 µm); Hamamatsu Orca-Flash 4.0 CMOS 6.5 µm	8 keV monochromatic; *t*_exp_ = 600 ms; projections = 1800; voxel size = 52 nm
spring 8: bl 47xu - 8kev - abs - 22.5 nm	SPring-8 BL47XU	Absorption contrast	Phase ring; BeamMonitor3 (Obj. lens ×10, LuAG 100 µm); Hamamatsu Orca-Flash 4.0 CMOS 6.5 µm	8 keV monochromatic; *t*_exp_ = 1000 ms; projections = 1800; voxel size = 18.45 nm
spring 8: bl 47xu - 8kev - pha - 18.45 nm	SPring-8 BL47XU	Zernike phase contrast	Phase ring; BeamMonitor3 (Obj. lens ×10, LuAG 100 µm); Hamamatsu Orca-Flash 4.0 CMOS 6.5 µm	8 keV monochromatic; *t*_exp_ = 1000 ms; projections = 1800; voxel size = 18.45 nm
esrf: id16b - 29.5kev - 4d (p=250) - 50.0 nm	ESRF ID16B	Holotomography / phase contrast	Phase ring; pco.edge 4.2 sCMOS 6.5 µm; Olympus Obj. lens ×10; LSO:Tb 30 µm	29.6 keV monochromatic; *t*_exp_ = 10 ms; projections = 2298; voxel size = 50 nm
esrf: id16B - 29.5kev - 4d (p=250) - 25.0 nm	ESRF ID16B	Holotomography / phase contrast	Phase ring; pco.edge 4.2 sCMOS 6.5 µm; Olympus Obj. lens ×10; LSO:Tb 30 µm	29.6 keV monochromatic; *t*_exp_ = 10 ms; projections = 2298; voxel size = 25 nm
esrf: id16b - 17.5kev - 3d (p=300) - 25.0 nm	ESRF ID16B	Holotomography / phase contrast	Phase ring; pco.edge 4.2 sCMOS 6.5 µm; Olympus Obj. lens ×10; LSO:Tb 30 µm	17.5 keV monochromatic; *t*_exp_ = 10 ms; projections = 2298; voxel size = 25 nm
esrf: id16b - 29.5kev - 2d (p=250) - 15.0 nm	ESRF ID16B	Holotomography / phase contrast	Phase ring; pco.edge 4.2 sCMOS 6.5 µm; Olympus Obj. lens ×10; LSO:Tb 30 µm	29.6 keV monochromatic; *t*_exp_ = 10 ms; projections = 2298; voxel size = 15 nm
desy: p05 - 11kev - pha - 56.2 nm	DESY PETRA III P05	Holotomography / phase contrast	Phase ring; Hamamatsu C12849-101U 6.5 µm	11 keV monochromatic; *t*_exp_ = 500 ms; projections = 718; voxel size = 56.2 nm

**Table 3:** SNR evaluated at 250 nm and 125 nm

Scan acronym	SNR_250nm_ (*h*^−1^)	SNR_125nm_ (*h*^−1^)
desy: p05 - 11kev - pha - 56.2 nm	23.6002	
esrf: id16b - 29.5kev - 2d (p=250) - 15.0 nm	862574.8726	25230.9647
esrf: id16b - 29.5kev - 4d (p=250) - 25.0 nm	4161.2210	160.0710
esrf: id16b - 17.5kev - 3d (p=300) - 25.0 nm	2468.5774	115.2457
esrf: id16b - 29.5kev - 4d (p=250) - 50.0 nm	159.9409	
spring 8: bl 47xu - 8kev - abs - 22.5 nm	133.5749	3.6601
spring 8: bl 47xu - 8kev - abs - 53.0 nm	0.5825	
spring 8: bl 47xu - 8kev - pha - 18.45 nm	197.0570	7.8129
spring 8: bl 47xu - 8kev - pha - 52.0 nm	2.8342	
spring 8: bl 20xu - 30kev - pha - 38.0 nm	33.6953	0.0120
fau: xradia 810 ultra - pha - 16.06 nm	11.9752	0.6320
u-saar: xrm-ii nanoct - pt-bt - 30.0 nm	0.0916	
u-saar: xrm-ii nanoct - w-nt - 20.0 nm	1.6775	0.0669
esrf: id16b - 29.5kev - 3d (p=250) - 25.0 nm	4668.4773	262.7617
esrf: id16b - 29.5kev - 4d (p=1200) - 25.0 nm	2679.8493	118.9089
esrf: id16b - 29.5kev - 3d ctf - 25.0 nm	10468.3052	669.5847

**Table 4:** MTF values at 10% and for comparison ESF transforms at 10%

Scan acronym	MTF 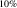 (nm)	MTF 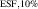 (nm)
desy: p05 - 11kev - pha - 56.2 nm	217.6116	166.31
esrf: id16b - 29.5kev - 2d (p=250) - 15.0 nm	35.2588	67.69
esrf: id16b - 29.5kev - 4d (p=250) - 25.0 nm	75.6818	165.44
esrf: id16b - 17.5kev - 3d (p=300) - 25.0 nm	78.2915	132.47
esrf: id16b - 29.5kev - 4d (p=250) - 50.0 nm	177.1277	154.52
spring 8: bl 47xu - 8kev - abs - 22.5 nm	66.6988	162.59
spring 8: bl 47xu - 8kev - abs - 53.0 nm	430.4634	123.83
spring 8: bl 47xu - 8kev - pha - 18.45 nm	63.7770	112.69
spring 8: bl 47xu - 8kev - pha - 52.0 nm	240.5000	137.42
spring 8: bl 20xu - 30kev - pha - 38.0 nm	115.0364	114.66
fau: xradia 810 ultra - pha - 16.06 nm	45.3219	65.46
u-saar: xrm-ii nanoct - pt-bt - 30.0 nm	170.2841	163.95
u-saar: xrm-ii nanoct - w-nt - 20.0 nm	55.6546	96.82
esrf: id16b - 29.5kev - 3d (p=250) - 25.0 nm	73.4559	109.05
esrf: id16b - 29.5kev - 4d (p=1200) - 25.0 nm	79.2857	128.69
esrf: id16b - 29.5kev - 3d ctf - 25.0 nm	59.0426	78.07

**Table 5:** Data processing parameters for different configurations

Scan acronym	Filters
desy: P05 - 11kev - pha - 56.2 nm	Gaussian (1, 30); local threshold (17.0); dilation (1); fill holes (8)
esrf: id16b - 29.5kev - 2d (p=250) - 15.0 nm	Gaussian (2, 15); curvature flow (10, 0.1); Otsu threshold; fill holes (20)
esrf: id16b - 29.5kev - 4d (p=250) - 25.0 nm	Gaussian (2, 15); curvature flow (10, 0.1); Otsu threshold; fill holes (20)
esrf: id16b - 29.5kev - 4d (p=250) - 50.0 nm	Gaussian (1); curvature flow (5, 0.1); local threshold (41); fill holes (20)
fau: xradia 810 ultra - pha - 16.06 nm	Gaussian (1, 5); Otsu threshold; fill holes (31)
spring 8: bl 47xu - 8kev - abs - 22.5 nm	Gaussian (1, 30); curvature flow (5, 0.1); Gaussian (2, 5); Otsu threshold; dilation (1); fill holes (27)
spring 8: bl 47xu - 8kev - abs - 53.0 nm	Gaussian (1, 30); local threshold (19.0); dilation (1); fill holes (9)
spring 8: bl 47xu - 8kev - pha - 18.45 nm	Gaussian (1, 10); curvature flow (5, 0.05); Gaussian (1, 15); Otsu threshold; fill holes (27)
spring 8: bl 47xu - 8kev - pha - 52.0 nm	Gaussian (0.5, 3); local threshold (33); fill holes (9)
spring 8: bl 20xu - 30kev - pha - 38.0 nm	Gaussian (1.5, 10); Otsu threshold; dilation (3); fill holes (13)
u-saar: xrm-ii nanoct - pt-bt - 30.0 nm	Gaussian (4.0, 20); local threshold; dilation (3); fill holes (13)
u-saar: xrm-ii nanoct - w-nt - 20.0 nm	Gaussian (3.0, 20); Otsu threshold

## Data Availability

The data analysis in this study was carried out using a GitLab CI pipeline, which automated the evaluation process and ensured reproducibility of the results. All relevant materials, including the raw data, processed data, figures, tables, and the code used for data processing, are openly available in the GitLab repository of the Deggendorf Institute of Technology: https://mygit.th-deg.de/roboct-public/sphereevaluation. The exact version of the data and code used in this work is archived under the tag https://mygit.th-deg.de/roboct-public/sphereevaluation/-/tree/paper?ref_type=tagspaper in the repository.

## Footnotes

1 The full evaluation pipeline and data are available at https://mygit.th-deg.de/roboct-public/sphereevaluation.

## Appendix A ESRF: algorithm study

While this study cannot judge the different algorithms used on the raw images, *e.g.* for retrieving an object’s transmission phase-map or reconstructing volume images of the latter, we could test at least one: the phase-retrieval algorithm employed by the ESRF ID16B. The core of this algorithm is the so-called Paganin filter which applies as Fourier de-convolution  to the raw images,

with *u* =  the radial amplitude of the image’s spatial frequencies *u*_*x*_ and *u*_*y*_ after Fourier-transforming and *p* commonly referred to as ‘Paganin’s parameter’. The latter scales with *d* and is commonly defined manually in order to yield the best compromise between de-convolution strength and low-pass filtering the images (a larger value of *p* yielding a stronger effect). Since a holotomography scan comprises multiple (4) congruent radiographs taken at different propagation distances *d*_0_, *d*_1_, *d*_2_ and *d*_3_, *p* is scaled accordingly while only one value is set by the user (for the shortest *d*_0_, hence the highest object magnification) (Yu *et al.*, 2018 ▸). Alternatively, one may choose a different phase retrieval algorithm, using the so-called contrast transfer function (CTF) which similarly combines images taken at multiple propagation distances from the object but requires manual settings only for regularization constants (Zabler *et al.*, 2005 ▸).

Fig. 14 ▸ shows the above reported MTF model fits for ‘esrf: id16b - 29.6kev 15 nm’, ‘25 nm’ and ‘50 nm’ which were all processed with Paganin’s parameter *p* = 250 µm but with different sets of propagation distances. The ‘15 nm’ scan comprised only two distances, the ‘25 nm’ scan four (but three when repeated at 17.5 keV) and the ‘50 nm’ four as well. The reason why a different number of distances was used each time was that a numerical alignment of the corresponding images’ coordinates and scaling is imperative for their correct superposition. In some cases images from one or two distances would not align well with the master-image (*d*_0_) causing strong blur. The problem is solved by leaving the ‘not well aligned’ distances out of the calculus and processing only those distances which align well.

Fig. 14 ▸ therefore shows additional model fits for the ‘29.6kev - 25 nm’ dataset, one using three instead of four distances (with *p* = 250 µm), the other one with all four distances but a higher parameter *p* = 1200 µm. Furthermore, the set of three distances was processed by CTF instead of Paganin’s de-convolution for comparing these two approaches. For completion, detection effectiveness (DE) is shown for the same cases in Fig. 15 ▸.

Leaving out one of four distances surprisingly yields an improvement both in MTF and DE, even if this gain is minor. The opposite (slightly less DE and slightly worse resolution) occurs when *p* is raised from 250 to 1200 µm. When CTF is employed (on three distances) instead of 10, the gain in resolution is more apparent. Visually, however, the result features stronger pixel noise compared with Fig. 8 ▸(*j*). Unlike the ‘25 nm’ scan, using CTF for the ‘50 nm’ or for the ‘15 nm’ scans yielded slightly worse resolution and/or worse SNR which is why we selected Paganin’s de-convolution with *p* = 250 µm as a baseline for this study.

In summary, testing and improving image processing routines which take action during nanoCT scans can be just as beneficial or detrimental as changing the experimental parameters. When system performance is reported, algorithms and soft-parameters must always be reported alongside.

## Appendix B Pipeline parameters

The parameters used in the computer-vision pipeline described in Section 2.5 for all datasets are listed in Table 5 ▸. These exact values are used in the GitLab CI pipeline to evaluate all reconstructed volume datasets.
